# Data on the genome and proteome profiles of ciprofloxacin-resistant *Acholeplasma laidlawii* strains selected under different conditions *in vitro*

**DOI:** 10.1016/j.dib.2020.106412

**Published:** 2020-10-19

**Authors:** Alexey Mouzykantov, Elena Medvedeva, Natalia Baranova, Victor Lopuhov, Konstantin Usachev, Olga Chernova, Vladislav Chernov

**Affiliations:** a Kazan Institute of Biochemistry and Biophysics, FRC Kazan Scientific Center of RAS, Russia; b Kazan (Volga Region) Federal University, Russia

**Keywords:** *Acholeplasma laidlawii*, Ciprofloxacin-resistant strains, Genomes, Proteomes

## Abstract

*Acholeplasma laidlawii* is widespread hypermutable bacteria (class Mollicutes) capable of infecting humans, animals, plants, which is the main contaminant of cell cultures and vaccine preparations. The mechanisms of the development of antimicrobial resistance of this bacterium are associated with the secretion of extracellular vesicles, which can mediate the lateral transfer of antibiotic resistance determinants. We compared the genome profiles of ciprofloxacin-resistant *A.laidlawii* strains PG8r1 (MIC 10 µg/ml) and PG8r3 (MIC 10 µg/ml) selected under different *in vitro* conditions - when ciprofloxacin-sensitive (MIC 0.5 µg/ml) *A.laidlawii* PG8B strain was cultured at increasing concentrations of ciprofloxacin in a broth medium alone, and with vesicles derived from the ciprofloxacin-resistant (MIC 20 µg/ml) *A.laidlawii* PG8R_10_c-2 strain, respectively. Genome profiles of PG8c-3 (obtained from a single colony of the strain PG8B) and PG8R_10_c-2 were analyzed too. Patterns of the quinolone target genes *(gyrA, gyrB, parE, parC*) containing in extracellular vesicles of PG8c-3, PG8R_10_c-2, PG8r1 and PG8r3 were determined. Genome sequencing was performed on the NextSeq Illumina platform. Search and annotation of single nucleotide polymorphisms were performed using Samtools and SnpEff, respectively. We also compared cellular proteomes of PG8c-3, PG8r1 and PG8r3. The cellular proteome profiles of the *A. laidlawii* strains were determined by two-dimensional gel electrophoresis and MALDI-TOF/TOF MS. This work presents data on single nucleotide polymorphisms (SNPs) found in the genomes of the ciprofloxacin-resistant strains selected under different *in vitro* conditions and proteins that were differentially expressed in the cells of ciprofloxacin-resistant strains selected under different conditions *in vitro*.

## Specifications Table

**Table:** 

Subject	Molecular biology
Specific subject area	Mollicute genomics and proteomics; antibiotic resistance
Type of data	Table
How data were acquired	Instruments: NextSeq Illumina platform, 2D gel electrophoresis, MALDI-TOF/TOF mass spectrometer Ultraflex III BRUKER Software: assembler SPAdes; Bowtie2; Samtools, Mascot Peptide Fingerprint
Data format	Raw Analyzed
Parameters for data collection	*A. laidlawii* strains with differential susceptibility to ciprofloxacin
Description of data collection	Three ciprofloxacin-resistant *Acholeplasma laidlawii* strains were selected under different conditions *in vitro*: sequentially inoculated in a broth medium that contained increasing concentrations of ciprofloxacin, co-culturing of ciprofloxacin-sensitive strain with and without extracellular vesicles derived from the high-level ciprofloxacin-resistant mollicute. The vesicles of *A. laidlawii* were obtained by ultracentrifugation; their purity was validated by PCR. Genome sequencing was performed on the NextSeq Illumina platform. Search and annotation of single nucleotide polymorphisms were performed using Samtools and SnpEff, respectively. Cellular proteins were separated by 2DE. Search of differential expressed proteins was performed using PDQuest. Proteins were identified by mass spectrometry.
Data source location	Institution: KIBB FRC Kazan Scientific Center of RAS, City/Town/Region: Kazan Country: Russia
Data accessibility	Repository name: GenBank Data identification number: [JACAOE000000000.1, JACAOF000000000.1, JACAOG000000000.1, JACAOH000000000.1] Direct URL to data: [https://www.ncbi.nlm.nih.gov/nuccore/JACAOE000000000.1, https://www.ncbi.nlm.nih.gov/nuccore/JACAOF000000000.1, https://www.ncbi.nlm.nih.gov/nuccore/JACAOG000000000.1, https://www.ncbi.nlm.nih.gov/nuccore/JACAOH000000000.1]

## Value of the Data

• These data document SNPs found in the genomes of ciprofloxacin-resistant *Acholeplasma laidlawii* strains, which were selected under different conditions *in vitro.*
• These data document proteins that were differentially expressed in the cells of ciprofloxacin-resistant *A. laidlawii* strains, which were selected under different conditions *in vitro.*
• These data may help to investigate different ways of developing ciprofloxacin resistance in *A. laidlawii.*
• These data may be useful for identification of effective drug targets to eliminate the mycoplasma contamination.

## 1 Data Description

**Fig. 1:**
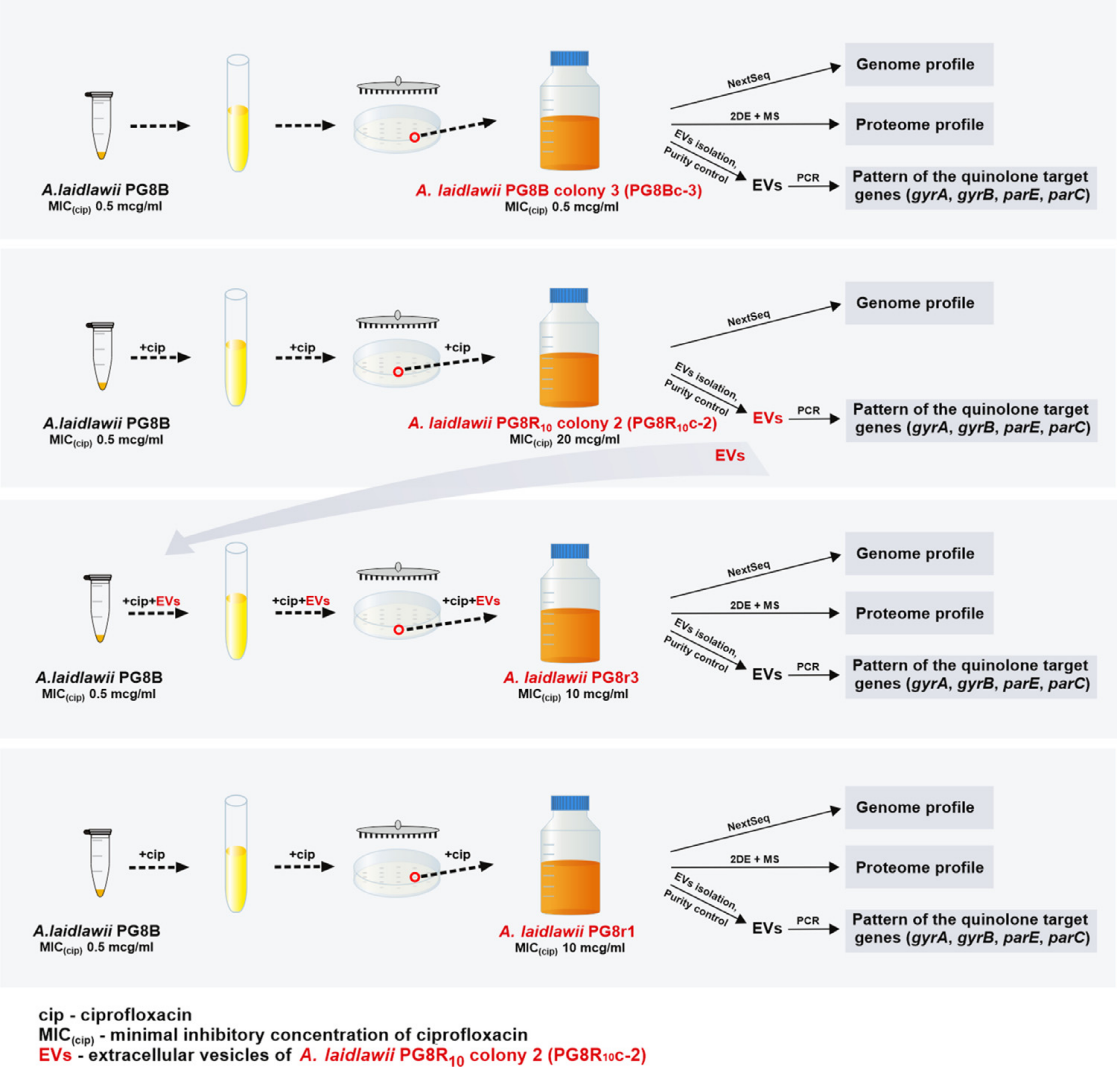
The scheme for obtaining ciprofloxacin-resistant *A.laidlawii* strains under different conditions *in vitro* and the data analysis steps.

**Fig. 2:**
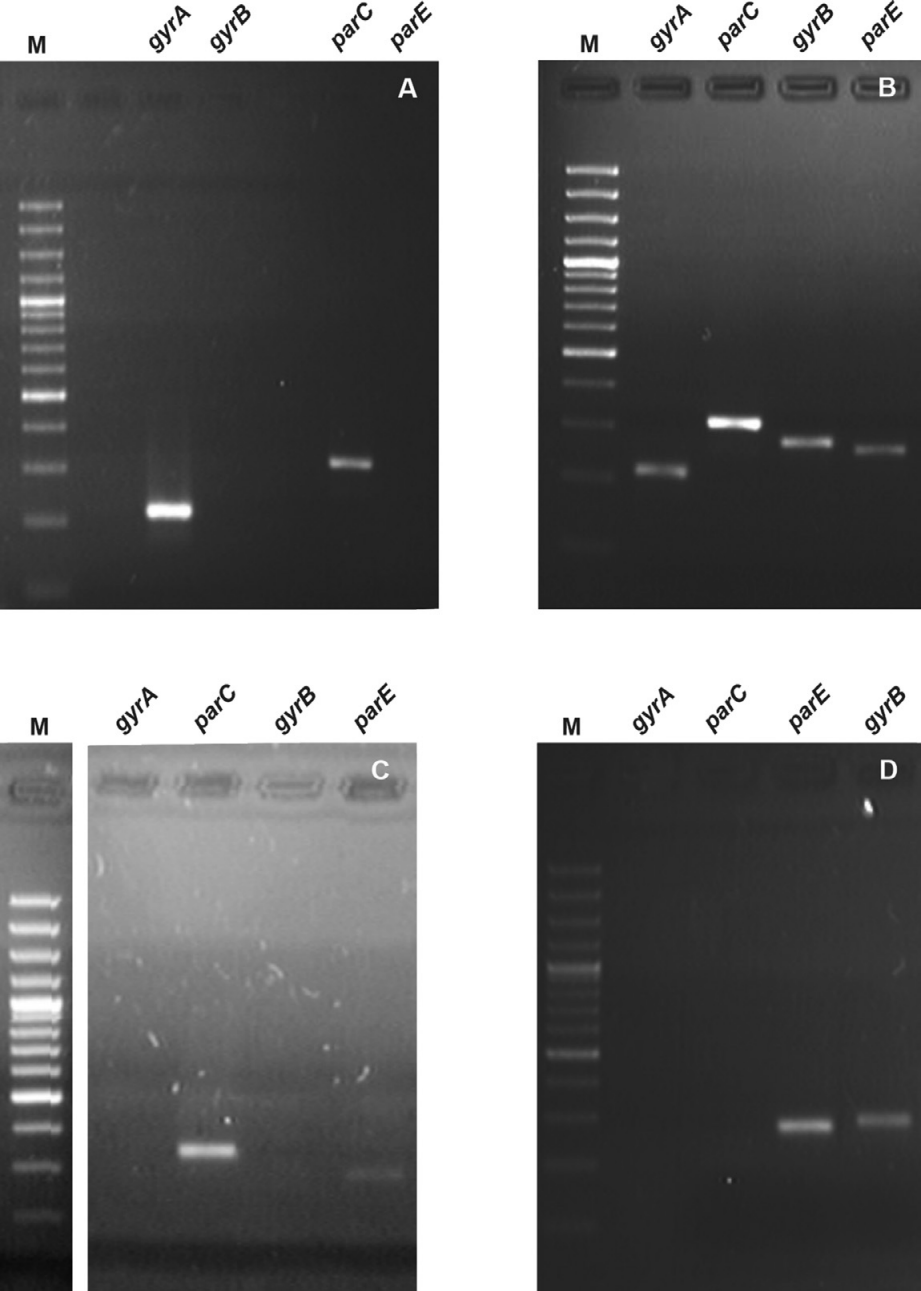
Electrophoregrams of the amplification products of the nucleotide sequences of *gyrA, gyrB, parE* and *parC* of *Acholeplasma laidlawii*, which were obtained by PCR using the total DNA (as a template) isolated from the EVs of ciprofloxacin-resistant *A.laidlawii* strains selected under different conditions *in vitro*. M - DNA Ladder Marker.

**Fig. 3:**
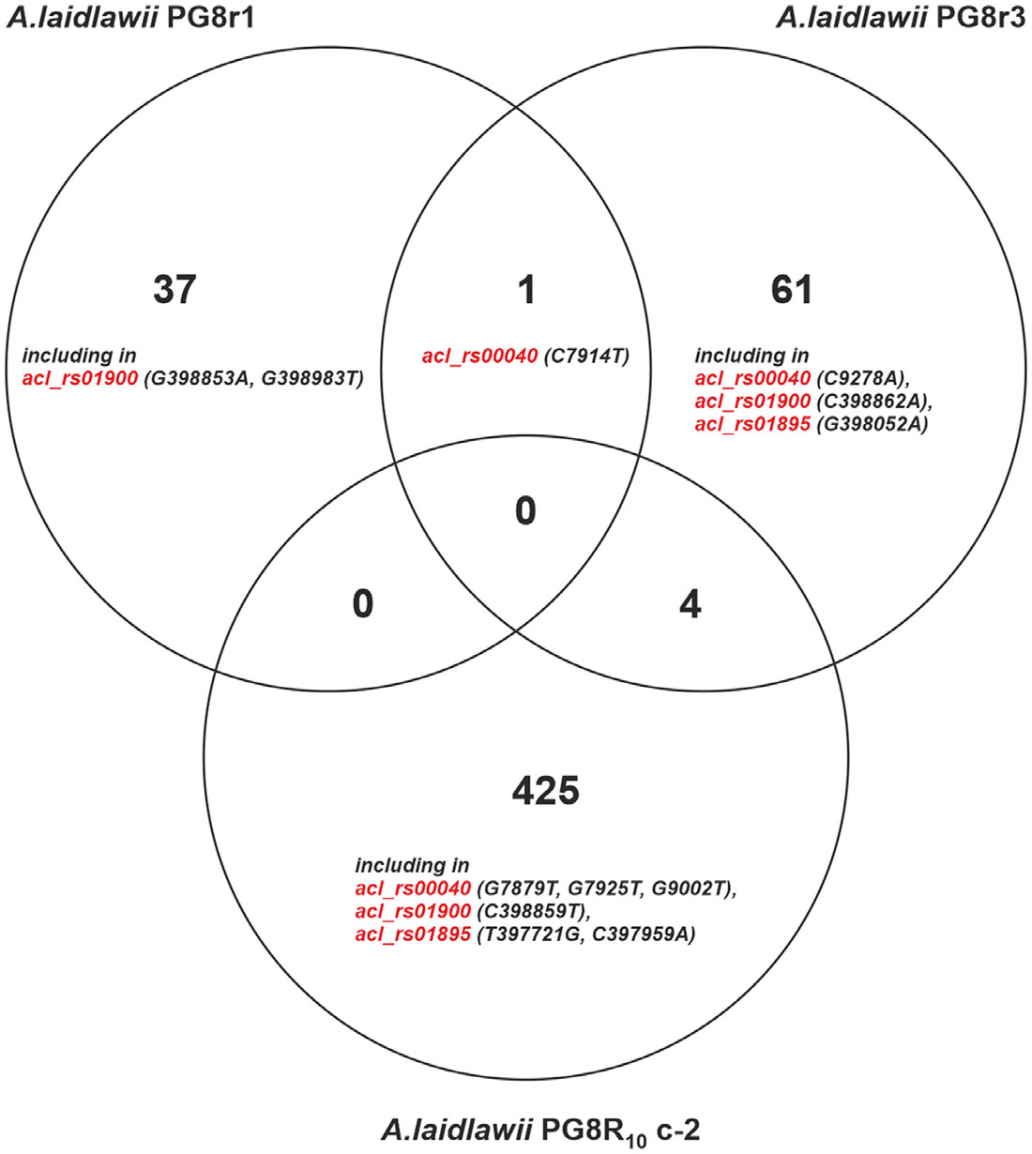
Venn diagram of common and specific SNPs in the genomes of ciprofloxacin-resistant *A.laidlawii* strains selected under different conditions *in vitro*. SNPs in genes encoding quinolone target proteins are indicated.

**Fig. 4:**
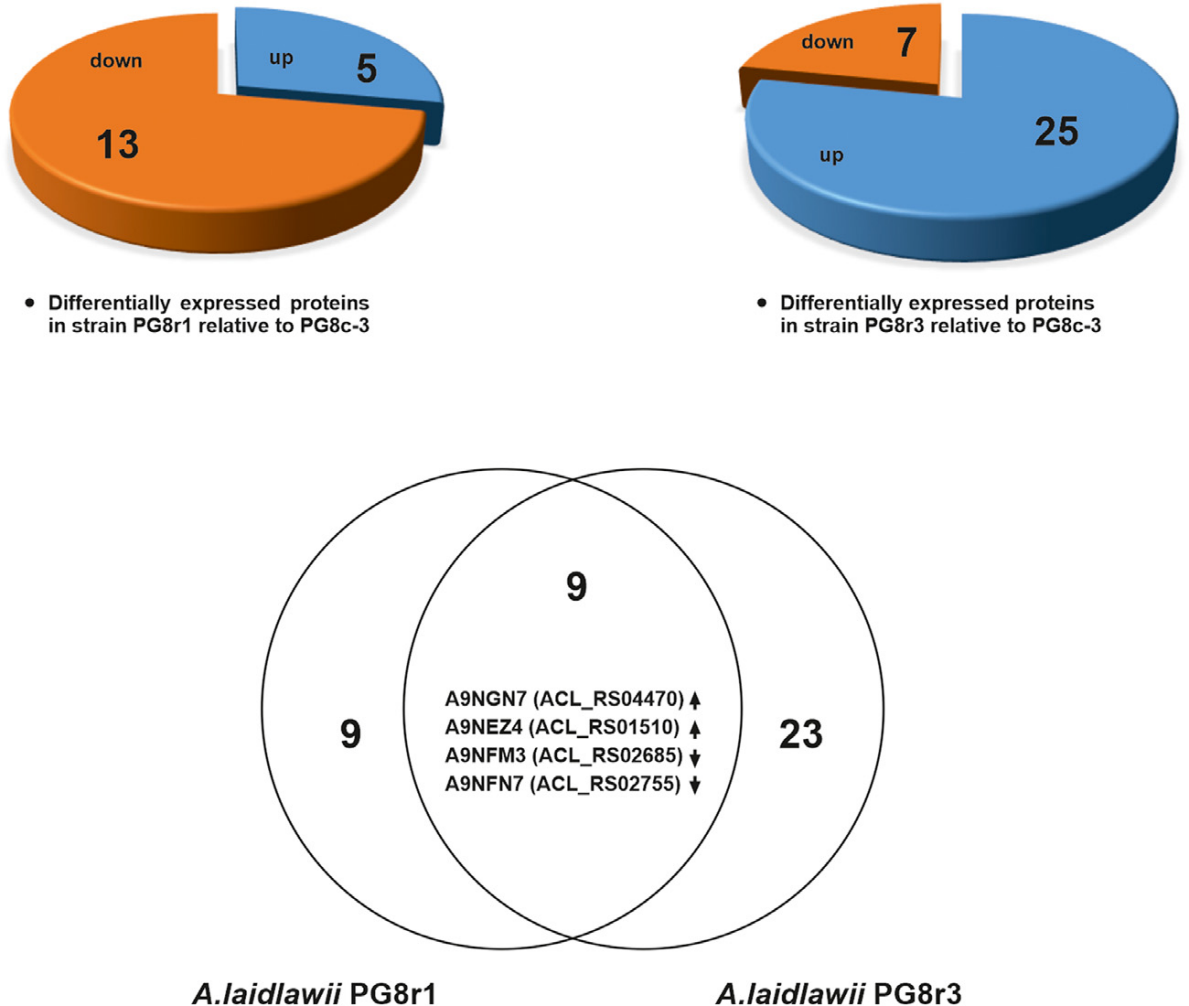
Venn diagram of common and specific proteins differentially expressed in ciprofloxacin-resistant *A.laidlawii* strains selected under different conditions *in vitro*. Common proteins with the similar character of changes are indicated.

This paper presents data on the features of the SNP profiles, as well as a features of patterns of differentially expressed proteins of ciprofloxacin-resistant *A. laidlawii* strains selected under different conditions *in vitro*. Genome profiles of the ciprofloxacin-resistant *A. laidlawii* strains PG8r1 (MIC 10 µg/ml) and PG8r3 (MIC 10 µg/ml) arising respectively when the ciprofloxacin-sensitive (MIC 0.5 µg/ml) *A. laidlawii* PG8B strain was cultured at increasing concentrations of ciprofloxacin in a broth medium alone and with vesicles derived from the ciprofloxacin-resistant (MIC 20 µg/ml) *A. laidlawii* PG8R_10_c-2 strain, were compared. Genome profiles of PG8c-3 (obtained from a single colony of the strain PG8B) and PG8R_10_c-2 were analyzed too (Fig. 1). Genomes of *A. laidlawii* strains are available in the GenBank database (accession numbers: JACAOE000000000.1, JACAOF000000000.1, JACAOG000000000.1 and JACAOH000000000.1). Patterns of the quinolone target genes *(gyrA, gyrB, parE, parC*) containing in extracellular vesicles of PG8c-3, PG8R_10_c-2, PG8r1 and PG8r3 were determined (Figs. 1 and 2, Supplementary Table 1). Single nucleotide polymorphisms (SNPs) in the genes encoding ciprofloxacin target proteins and beyond ones were registered in the ciprofloxacin-resistant strains selected under different condition *in vitro*. Common and specific SNPs are highlighted (Fig. 3). Lists of identified SNPs in the genomes of ciprofloxacin-resistant *A. laidlawii* strains are presented in Supplementary Tables 2–4. Cellular proteomes of PG8c-3, PG8r1 and PG8r3 were compared (Fig. 1). The cellular proteome profiles of the *A. laidlawii* strains were determined by two-dimensional gel electrophoresis and MALDI-TOF/TOF MS. Proteins that were differentially expressed in the cells of ciprofloxacin-resistant strains selected under different conditions *in vitro* were identified. Proteins with the similar character of change are highlighted (Fig. 4). Lists of differentially expressed proteins in ciprofloxacin-resistant *A. laidlawii* strains are presented in Supplementary Tables 5, 6.

A – DNA isolated from EVs of *A. laidlawii* PG8Bc-3 strain B – DNA isolated from EVs of *A. laidlawii* PG8R_10_c-2 strain C – DNA isolated from EVs of *A. laidlawii* PG8r1 strain D – DNA isolated from EVs of *A. laidlawii* PG8r3 strain.

## 2 Experimental Design, Materials and Methods

### 2.1 Bacterial strains and culture conditions

Cells of ciprofloxacin-resistant strain *A. laidlawii* PG8R_10_c-2 (MIC 20 μg/ml) were cultivated in Edward's medium (tryptose 2%; NaCl 0.5%; KCl 0.13%; Tris-base 0.3%; horse blood serum 10%; yeast extract 5%; glucose 1%; penicillin 1000 U/ml; phenol red 0.3 ml of 1% solution) in the presence of ciprofloxacin (10 μg/ml). The minimum inhibitory concentration (MIC) of the cultures was determined using the dilution method in a liquid nutrient medium with various antibiotic concentrations [1]. Cultivation of *A. laidlawii* PG8B with vesicles of *A. laidlawii* PG8R_10_c-2 was carried out according to [1] with modifications in triplicates. *A. laidlawii* PG8B was cultivated in Edward's medium at 37 °C until the middle of the logarithmic phase. Then, the cells were pelleted by centrifugation, suspended in Edward's medium to a concentration of 10^7^ cells/ml, and vesicles (15–20 µg/ml protein) isolated from PG8R_10_c-2 strain were added along with ciprofloxacin at a concentration of 0.5 µg/ml. Suspensions were incubated for 6 h at 37 °C, then Edward's medium was added to each suspension and they were incubated at 37 °C until the middle of the log phase. This scheme was repeated, with an increase in the concentration of the antibiotic in each cycle, to ciprofloxacin concentration of 10 μg/ml. The ciprofloxacin-resistant (MIC 10 µg/ml) strain resulting from this procedure was designated PG8r3. The ciprofloxacin-resistant (MIC 10 μg/ml) PG8r1 strain was obtained similarly, but without the addition of PG8R_10_c-2 vesicles. Patterns of the quinolone target genes *(gyrA, gyrB, parE, parC*) containing in extracellular vesicles of PG8c-3, PG8R_10_c-2, PG8r1 and PG8r3 were determined (Supplementary Table 1).

### 2.2 Extraction and purification of extracellular vesicles

The isolation of the *A. laidlawii* PG8R_10_c-2 extracellular vesicles was performed according to [2]. The cells were removed from the culture broth by centrifuging at 6000 g for 20 min, after which any residual cells were removed from the supernatant by filtration using 0.1 μm PES filter (Sartorius). Supernatant was concentrated using 100 kDa Vivacell 100 (Sartorius, Germany). The vesicles were pelleted by ultracentrifugation at 100,000 g, 1 h, 8 °C (Beckman Coulter Optima™MAX-E). Crude EVs preparation was then resuspended in buffer (50 mM Tris-HCl, pH 7.4; 150 mM NaCl; 2 mM MgCl_2_) and placed on a stepwise density gradient 20%–40% Optiprep (Sigma) and ultracentrifuged (100,000 g, 3 h, 8 °C). The vesicular fraction was collected, diluted threefold in buffer and then ultracentrifuged again. The pellet was resuspended in buffer supplemented with 1 mM PMSF (Fluka) and stored at 8 °C. The absence of microbial cells in the vesicle preparation was tested, plating on Edward's medium and PCR analysis with primers for marker nucleotide sequences of vesicle – 16S-23S rRNA gene intergenic spacer region^−^, *ftsZ^−^, pnp*^+^, *tufB*^+^.

### 2.3 Polymerase chain reaction

Vector NTI 9.1.0 (Invitrogen) was used to design the primers for amplification and synthesized in Litekh Research and Production Company (Moscow, Russia): *ftsZ* (5′-ggtttttggatttaacgatg-3′ and 5′-gcttccgcctcttttattt-3′), 16S–23S rRNA gene intergenic spacer region (5′-ggaggaaggtggggatgacgtcaa-3′ and 5′-ccttaggagatggtcctcctatcttcaaac-3′), *pnp* (5′-aagcccattgcgatacctgc-3′ and 5′-ggtgctttaggagaacgtgct-3′), *tufB* (5′-ccaggtcacgctgactatgtt-3′ and 5′-acgagtttgtggcattggac-3′), *gyrA* (5’-atcagcgagaatcgttgg-3’ and 5’-tctctaaccatttcaccagc-3’), *parC* (5’-atacgcaatgggacaaatg-3’ and 5’-ggttcttgttcctcatcatca-3’), *gyrB* (5’-gtaaactagcggattgcc-3’ and 5’-tcagcatcggtcataataac-3’), *parE* (5’-tgctcaaggtaaagataaatca-3’ and 5’-cggcatcagtcataataatca-3’). PCR reactions were performed in 25 μl volumes. The thermal profile was as follows: for *ftsZ* 95 °C, 3 min; followed by 30 cycles of [95 °C, 30 s; 52 °C, 90 s; 72 °C, 60 s] and a final extension at 72 °C, 10 min; for 16S–23S rRNA gene intergenic spacer region, 95 °C, 3 min; followed by 30 cycles of [95 °C, 5 s; 63 °C, 5 s; 72 °C, 20 s] and a final extension at 72 °C, 5 min; for *pnp, tufB, gyrA, gyrB, parE* and *parC* 95 °C, 3 min; followed by 35 cycles of [95 °C, 5 s; 52 °C 5 s; 72 °C 5 s] and a final extension at 72 °C 5 min. PCR products were separated on a 2% agarose gel by electrophoresis and then stained with ethidium bromide.

### 2.4 DNA preparation and sequencing

DNA was isolated from the cells of strains (*A. laidlawii* PG8Bc-3, PG8R_10_c-2, PG8r1, and PG8r3), using the phenol extraction method with additional treatment with proteinase K and RNase [3]. Whole genome sequencing was performed on the NextSeq Illumina platform (USA). DNA was fragmented enzymatically using the NEBNext Ultra II FS DNA Library Prep Kit for Illumina. Libraries were created from the obtained DNA fragments according to the manufacturer's instructions. The quality of the resulting libraries was evaluated using a 2100 Bioanalyzer instrument (Agilent Technologies). DNA concentration was determined using a Qubit 2.0 fluorimeter (Invitrogen).

### 2.5 Proteins preparation

Proteins from cells of *A. laidlawii* were isolated as described previously [2]. The *A. laidlawii* cells were pelleted (6000 g, 20 min) and washed twice with buffer (150 mM NaCl, 50 mM Tris, 2 mM MgCl_2_.6H_2_O, pH 7.4) and once in the same buffer with PMSF. The pellet of cells was treated with CHAPS and Micrococcal Nuclease Mix (Thermo Fisher Scientific, USA). The resulting proteins were dissolved in a solution containing 8 M urea, 2 M thiourea, 5% ampholines (pH 3–10), 80 mM dithiothreitol (DTT), 5% CHAPS and 1.67% NP-40. The protein concentration in the samples was measured by the Bradford method.

### 2.6 2D-PAGE and gel analysis

Proteins were separated using 2DE as described previously [4]. Isoelectrofocusing (IEF) was performed in glass tubes in 4% polyacrylamide gel (8 M urea, 4% acrylamide/bis-acrylamide, 1.75% ampholines (pH 3–10), 3.5% ampholines (pH 5–8), 1.8% CHAPS and 0.6% NP-40, 0.1% TEMED, 0.02% PSA). IEF was done in the following regime: 100 V-200 V-300 V-400 V-500 V-600 V for 45 min, 700 V for 10 h, 900 V for 2 h. Prior to 2nd dimension gels were equilibrated once with equilibration buffer (6 M urea, 30% glycerol, 62.5 mM Tris-HCl (pH 6.8), 2% SDS, bromophenol blue, 20 mM DTT) at room temperature for 15 min. Second-dimension separation was performed using Protean II xi Cell electrophoresis system (Bio-Rad) using 12% SDS-PAGE. Electrophoresis was performed in Tris-glycine buffer (25 mM Tris, 192 mM glycine, pH 8.3) at the following regime: 40 mA for 20 min, 80 mA for 2 h, 70 mA for 2.5 h. The gels were stained with Coomassie Brilliant Blue G-250 [5]. The gels were scanned and analyzed with PDQuest (ver. 8.0.1) software (Bio-Rad Laboratories, Inc., USA). Spots that were present in all three replicates were selected for subsequent comparison and identification. A cutoff value was set at a 1.5-fold increase or decrease.

### 2.7 Tryptic digestion of proteins

Proteins were extracted from the gel and hydrolyzed using the protocol described in [6]. The protein spots were cut out from the gel and washed in ddiH_2_O and mix of acetonitrile: 200 mM NH_4_HCO_3_ (1:1) at 50 °C 30 min. Protein reduction was performed using 10 mM DTT 100 mM NH_4_HCO_3_ for 1 h, followed by alkylation using a mixture of 50 mM iodoacetamide and 100 mM NH_4_HCO_3_ in the dark for 45 min at room temperature. The gels were incubated in acetonitrile, dried and incubated in trypsin Gold (Promega) solution for 60 min at 4 °C. Tryptic digestion was performed at 37 °C overnight. To extract peptides, a solution containing 0.1% trifluoroacetic acid in deionized water was added to gel fragments that were then incubated in an ultrasonic bath for 10 min. The resulting supernatants were sampled into separate tubes.

### 2.8 Protein identification by MALDI TOF/TOF MS

The identification of proteins was performed using MALDI-TOF/TOF mass spectrometer Ultraflex III BRUKER (USA) with a UV-laser in the positive ion mode in the diapason of 500–4000 Da using reflectron [7]. The proteins were identified from the masses of proteolytic fragments using Mascot Peptide Mass Fingerprint (Matrix Science, USA) software and UniProt database. Searches were performed allowing up to 1 trypsin miscleavage. Variable modifications included carbamidomethylation of cysteine and oxidation of methionine. Peptide mass tolerance was set to 60 ppm. A protein score of ≥44 was considered a significant matched (*p* < 0.05).

### 2.9 Bioinformatics data analysis

The assembly of DNA sequences *de novo* was performed using assembler SPAdes. Nucleotide sequence alignment was performed using Bowtie2 (http://bowtie-bio.sourceforge.net/bowtie2/index.shtml), single nucleotide polymorphism (SNP) searches and annotations were performed using Samtools (http://samtools.sourceforge.net/mpileup.shtml) and SnpEff (http://snpeff.sourceforge.net/SnpEff. html), respectively [8,9].

## Ethics Statement

The work involved bacteria, but did not involve the use of human subjects or animals.

## Declaration of Competing Interest

The authors declare that they have no known competing financial interests or personal relationships which have, or could be perceived to have, influenced the work reported in this article.

## Appendix Supplementary materials

Image, application 1

Image, application 2

Image, application 3

Image, application 4

Image, application 5

Image, application 6
