# Supplementary material for: Data on the genome and proteome profiles of ciprofloxacin-resistant Acholeplasma laidlawii strains selected under different conditions in vitro
Source: Data Brief. 2020 Oct 19;33:106412. doi: 10.1016/j.dib.2020.106412 (PMC7585042; doi:10.1016/j.dib.2020.106412)
Supplement: Supplementary file 1 [file mmc1.docx]

**Supplementary table 1.** Pattern of genes encoding ciprofloxacin target proteins found in extracellular vesicles of *A.laidlawii* PG8Bc-3, *A.laidlawii* PG8R_10_c-2, *A.laidlawii* PG8r1 and *A.laidlawii* PG8r3 strains

| **Gene name** | **Gene locus** | **Protein name** | ***A. laidlawii*** | | | |
| --- | --- | --- | --- | --- | --- | --- |
|  |  |  | **PG8Bc-3** | **PG8R_10_c-2** | **PG8r1** | **PG8r3** |
| *gyrA* | ACL_RS00040 | DNA gyrase subunit A | **+** | **+ *** | **-** | **-** |
| *parC* | ACL_RS01900 | DNA topoisomerase IV subunit A | **+** | **+ *** | **+**** | **-** |
| *gyrB* | ACL_RS00035 | DNA topoisomerase (ATP-hydrolyzing) subunit B | - | **+** | - | **+** |
| *parE* | ACL_RS01895 | DNA topoisomerase IV subunit B | - | **+ *** | + | **+***** |

* SNPs in the *gyrA* gene (G7879T, G7925T and G9002T), *parC* gene (C398859T), *parE* gene (T397721G and C397959A) of the strain were found.

** SNPs in the *parC* gene (G398853A and G398983T) of the strain were found.

*** SNP in the *parE* gene (G398052A) of the strain was found.
