# Supplementary material for: Data on the genome and proteome profiles of ciprofloxacin-resistant Acholeplasma laidlawii strains selected under different conditions in vitro
Source: Data Brief. 2020 Oct 19;33:106412. doi: 10.1016/j.dib.2020.106412 (PMC7585042; doi:10.1016/j.dib.2020.106412)
Supplement: Supplementary file 2 [file mmc2.docx]

**Supplementary table 2.** SNPs in genome of *A. laidlawii* PG8r3

| № | Protein Name^1^ | Locus tag^2^ | Position^3^ | PG8B^4^ | PG8r3^5^ | Effect |
| --- | --- | --- | --- | --- | --- | --- |
|  | **Energy production and conversion** |  |  |  |  |  |
| 1 | Formate C-acetyltransferase | ACL_RS00145 | 34008 | G | T | stop gained |
| 2 | RnfABCDGE type electron transport complex subunit C | ACL_RS00245 | 58929 | G | T | stop gained |
| 3 | NAD(P)/FAD-dependent oxidoreductase | ACL_RS02335 | 492521 | G | T | non synonymous coding |
|  | **Cell cycle control, cell division, chromosome partitioning** |  |  |  |  |  |
| 4 | Septum formation protein Maf | ACL_RS06840 | 1445068 | G | T | non synonymous coding |
|  | **Amino acid transport and metabolism** |  |  |  |  |  |
| 5 | 3-deoxy-7-phosphoheptulonate synthase | ACL_RS01060 | 207894 | G | T | stop gained |
|  | **Nucleotide transport and metabolism** |  |  |  |  |  |
| 6 | Ribose-phosphate pyrophosphokinase | ACL_RS00095 | 21375 | C | A | non synonymous coding |
| 7 | Guanylate kinase | ACL_RS01520 | 314078 | A | C | non synonymous coding |
| 8 | Adenylosuccinate lyase | ACL_RS06595 | 1390927 | C | A | synonymous coding |
|  | **Carbohydrate transport and metabolism** |  |  |  |  |  |
| 9 | Ribulose-phosphate 3-epimerase | ACL_RS01245 | 253510 | C | A | synonymous coding |
| 10 | N-acetylglucosamine kinase | ACL_RS01390 | 293971 | G | T | non synonymous coding |
| 11 | Phosphoenolpyruvate-protein phosphotransferase | ACL_RS02185 | 458312 | C | A | non synonymous coding |
| 12 | Alpha-amylase | ACL_RS03330 | 699586 | C | A | non synonymous coding |
| 13 | sn-glycerol-3-phosphate ABC transporter ATP-binding protein UgpC | ACL_RS03335 | 701440 | C | A | synonymous coding |
|  | **Coenzyme transport and metabolism** |  |  |  |  |  |
| 14 | Biotin-[acetyl-CoA-carboxylase] ligase | ACL_RS07200 | 491493 | G | T | non synonymous coding |
|  | **Transcription** |  |  |  |  |  |
| 15 | DNA-directed RNA polymerase subunit beta' | ACL_RS00845 | 160197 | G | T | non synonymous coding |
|  | **Replication, recombination and repair** |  |  |  |  |  |
| 16 | DNA gyrase subunit A | ACL_RS00040 | 7914 | C | T | non synonymous coding |
| 17 | DNA gyrase subunit A | ACL_RS00040 | 9278 | C | A | non synonymous coding |
| 18 | Ribonuclease M5 | ACL_RS00080 | 19170 | G | T | non synonymous coding |
| 19 | Endonuclease | ACL_RS07130 | 140582 | C | T | non synonymous coding |
| 20 | Holliday junction branch migration DNA helicase RuvB | ACL_RS01850 | 389633 | C | A | non synonymous coding |
| 21 | DNA topoisomerase IV subunit B | ACL_RS01895 | 398052 | G | A | non synonymous coding |
| 22 | DNA topoisomerase IV subunit A | ACL_RS01900 | 398862 | C | A | non synonymous coding |
| 23 | ATP-dependent RecD-like DNA helicase | ACL_RS02810 | 598278 | C | A | non synonymous coding |
| 24 | Tyrosine-type recombinase/integrase | ACL_RS02915 | 618302 | G | T | non synonymous coding |
| 25 | HU family DNA-binding protein | ACL_RS04310 | 902547 | G | T | non synonymous coding |
|  | **Cell wall/membrane/envelope biogenesis** |  |  |  |  |  |
| 26 | Glycosyltransferase family 4 protein | ACL_RS02440 | 511524 | C | A | non synonymous coding |
|  | **Cell motility** |  |  |  |  |  |
| 27 | Leucine-rich repeat domain-containing protein | ACL_RS03825 | 807691 | C | A | non synonymous coding |
|  | **Posttranslational modification, protein turnover, chaperones** |  |  |  |  |  |
| 28 | Ribonuclease J | ACL_RS01545 | 320074 | C | A | stop gained |
|  | **Inorganic ion transport and metabolism** |  |  |  |  |  |
| 29 | ABC transporter substrate-binding protein | ACL_RS02370 | 498071 | G | T | start lost |
|  | **Signal transduction mechanisms** |  |  |  |  |  |
| 30 | 50S ribosomal protein L9 | ACL_RS00960 | 187588 | C | A | non synonymous coding |
| 31 | Arsenate reductase ArsC | ACL_RS02455 | 513306 | C | A | non synonymous coding |
| 32 | Bifunctional diguanylate cyclase/phosphodiesterase | ACL_RS05575 | 1191063 | C | A | stop gained |
|  | **Defense mechanisms** |  |  |  |  |  |
| 33 | MATE family efflux transporter | ACL_RS02470 | 517270 | C | A | non synonymous coding |
| 34 | AAA family ATPase | ACL_RS07235 | 657577 | C | A | non synonymous coding |
| 35 | ABC transporter ATP-binding protein | ACL_RS04180 | 878248 | T | G | non synonymous coding |
| 36 | Hypothetical protein | ACL_RS05520 | 1154235 | G | T | non synonymous coding |
|  | **Function unknown** |  |  |  |  |  |
| 37 | Coenzyme F420-0:L-glutamate ligase | ACL_RS00110 | 26114 | C | A | non synonymous coding |
| 38 | DegV family protein | ACL_RS01055 | 206468 | G | T | non synonymous coding |
| 39 | Phage major capsid protein | ACL_RS03055 | 643769 | G | T | stop gained |
| 40 | Hypothetical protein | ACL_RS03090 | 648520 | G | T | non synonymous coding |
| 41 | FAD-dependent oxidoreductase | ACL_RS03745 | 793355 | G | T | non synonymous coding |
| 42 | Hypothetical protein | ACL_RS04130 | 869487 | C | A | synonymous coding |
| 43 | InlB B-repeat-containing protein | ACL_RS05535 | 1173807 | G | T | non synonymous coding |
| 44 | InlB B-repeat-containing protein | ACL_RS05535 | 1178659 | T | G | non synonymous coding |
| 45 | InlB B-repeat-containing protein | ACL_RS05535 | 1181206 | G | T | non synonymous coding |
| 46 | GTPase HflX | ACL_RS06775 | 1431528 | G | T | non synonymous coding |
| 47 | Hypothetical protein | ACL_RS06970 | 1470488 | G | T | non synonymous coding |
|  | **Not in EggNog** |  |  |  |  |  |
| 48 | Hypothetical protein | ACL_RS00695 | 124003 | C | A | non synonymous coding |
| 49 | Peptide chain release factor N(5)-glutamine methyltransferase | ACL_RS00990 | 193545 | G | T | non synonymous coding |
| 50 | Membrane protein insertion efficiency factor YidD | ACL_RS01485 | 307672 | G | T | non synonymous coding |
| 51 | Hypothetical protein | ACL_RS02135 | 446643 | G | T | non synonymous coding |
| 52 | Hypothetical protein | ACL_RS02580 | 539511 | G | T | non synonymous coding |
| 53 | McrC family protein | ACL_RS03165 | 660810 | G | T | non synonymous coding |
| 54 | Energy-coupled thiamine transporter ThiT | ACL_RS04125 | 869008 | C | A | stop gained |
| 55 | Hypothetical protein | ACL_RS05300 | 1107845 | C | A | non synonymous coding |
| 56 | Hypothetical protein | ACL_RS05770 | 1220417 | C | T | non synonymous coding |
| 57 | Hypothetical protein | ACL_RS06250 | 1319624 | C | A | stop gained |
| 58 | Hypothetical protein | ACL_RS06630 | 1399171 | C | A | stop gained |
| 59 | Hypothetical protein | ACL_RS06965 | 1468886 | A | C | non synonymous coding |
|  | **Intergenic regions** |  |  |  |  |  |
| 60 |  |  | 4393 | G | T | intergenic |
| 61 |  |  | 73091 | G | T | intergenic |
| 62 |  |  | 837820 | G | T | intergenic |
| 63 |  |  | 902615 | G | T | intergenic |
| 64 |  |  | 1082307 | C | A | intergenic |
|  | **Pseudogenes** |  |  |  |  |  |
| 65 |  | ACL_RS00300 | 67020 | G | A | pseudo |
| 66 |  | ACL_RS06385 | 1348489 | G | T | pseudo |

^1^Name of protein encoded by mutant gene/functional category according to EggNOG; ^2^Gene locus on *A.laidlawii* chromosome according to GenBank; ^3^The position of the SNP in the nucleotide sequence of *A. laidlawii* PG8r3 strain; ^4^Nucleotide in *A.laidlawii* PG8Bc-3 strain; ^5^Nucleotide in *A. laidlawii* PG8r3 strain.
