# Supplementary material for: Data on the genome and proteome profiles of ciprofloxacin-resistant Acholeplasma laidlawii strains selected under different conditions in vitro
Source: Data Brief. 2020 Oct 19;33:106412. doi: 10.1016/j.dib.2020.106412 (PMC7585042; doi:10.1016/j.dib.2020.106412)
Supplement: Supplementary file 3 [file mmc3.docx]

**Supplementary table 3.** SNPs in genome of *A. laidlawii* PG8r1

| № | Protein Name^1^ | Locus tag^2^ | Position^3^ | PG8B^4^ | PG8r1^5^ | Effect |
| --- | --- | --- | --- | --- | --- | --- |
|  | **Energy production and conversion** |  |  |  |  |  |
| 1 | GTPase ObgE | ACL_RS01825 | 382823 | G | T | synonymous coding |
|  | Amino acid transport and metabolism |  |  |  |  |  |
| 2 | M3 family oligoendopeptidase | ACL_RS05050 | 1046387 | C | A | non synonymous coding |
| 3 | ABC transporter ATP-binding protein | ACL_RS06040 | 1268702 | C | A | non synonymous coding |
| 4 | Aspartate-ammonia ligase | ACL_RS06080 | 1279627 | G | T | non synonymous coding |
|  | **Nucleotide transport and metabolism** |  |  |  |  |  |
| 5 | Deoxynucleoside kinase | ACL_RS01955 | 411271 | G | T | non synonymous coding |
|  | Carbohydrate transport and metabolism |  |  |  |  |  |
| 6 | Glucose-1-phosphate adenylyltransferase | ACL_RS02605 | 544130 | G | T | non synonymous coding |
| 7 | Alpha-amylase | ACL_RS03285 | 686237 | C | A | non synonymous coding |
| 8 | Hypothetical protein | ACL_RS03615 | 768302 | C | A | non synonymous coding |
|  | **Coenzyme transport and metabolism** |  |  |  |  |  |
| 9 | FAD:protein FMN transferase | ACL_RS00275 | 64657 | C | A | synonymous coding |
|  | **Lipid transport and metabolism** |  |  |  |  |  |
| 10 | Phosphate acyltransferase PlsX | ACL_RS01115 | 220198 | G | T | non synonymous coding |
|  | **Translation, ribosomal structure and biogenesis** |  |  |  |  |  |
| 11 | Ribosomal L7Ae/L30e/S12e/Gadd45 family protein | ACL_RS01595 | 328130 | C | A | non synonymous coding |
| 12 | 23S rRNA (uracil(1939)-C(5))-methyltransferase RlmD | ACL_RS02855 | 604682 | C | T | non synonymous coding |
| 13 | Bifunctional oligoribonuclease/PAP phosphatase NrnA | ACL_RS06955 | 1466715 | C | A | synonymous coding |
|  | **Transcription** |  |  |  |  |  |
| 14 | DNA-directed RNA polymerase subunit beta' | ACL_RS00845 | 160074 | G | T | non synonymous coding |
| 15 | DNA-directed RNA polymerase subunit beta' | ACL_RS00845 | 161020 | C | A | non synonymous coding |
| 16 | NAD-dependent protein deacylase | ACL_RS01430 | 300665 | G | T | stop gained |
| 17 | LacI family DNA-binding transcriptional regulator | ACL_RS02660 | 563508 | G | T | synonymous coding |
| 18 | bifunctional (p)ppGpp synthetase/guanosine-3',5'-bis(diphosphate) 3'-pyrophosphohydrolase | ACL_RS04095 | 862629 | A | C | synonymous coding |
|  | **Replication, recombination and repair** |  |  |  |  |  |
| 19 | DNA gyrase subunit A | ACL_RS00040 | 7914 | C | A | non synonymous coding |
| 20 | Transcription-repair coupling factor | ACL_RS00105 | 24598 | G | T | non synonymous coding |
| 21 | DNA topoisomerase IV subunit A | ACL_RS01900 | 398853 | G | A | non synonymous coding |
| 22 | DNA topoisomerase IV subunit A | ACL_RS01900 | 398983 | G | T | non synonymous coding |
| 23 | DNA mismatch repair endonuclease MutL | ACL_RS04360 | 910648 | C | A | non synonymous coding |
| 24 | NUDIX domain-containing protein | ACL_RS04540 | 949941 | G | T | non synonymous coding |
| 25 | NAD-dependent DNA ligase LigA | ACL_RS06670 | 1408244 | C | A | non synonymous coding |
|  | **Function unknown** |  |  |  |  |  |
| 26 | SOS response-associated peptidase | ACL_RS02635 | 556535 | T | A | non synonymous coding |
| 27 | Hypothetical protein | ACL_RS03090 | 646930 | C | A | non synonymous coding |
| 28 | YIP1 family protein | ACL_RS03590 | 760955 | C | A | synonymous coding |
| 29 | C39 family peptidase | ACL_RS05510 | 1151163 | C | A | non synonymous coding |
| 30 | DUF1295 domain-containing protein | ACL_RS06185 | 1299986 | C | A | stop gained |
|  | **Not in EggNog** |  |  |  |  |  |
| 31 | Sensor domain-containing diguanylate cyclase | ACL_RS07115 | 118692 | G | T | non synonymous coding |
| 32 | Hypothetical protein | ACL_RS01325 | 276171 | G | A | non synonymous coding |
| 33 | Hypothetical protein | ACL_RS01830 | 384015 | G | T | non synonymous coding |
| 34 | Hypothetical protein | ACL_RS01910 | 403807 | G | T | non synonymous coding |
| 35 | McrC family protein | ACL_RS03165 | 660560 | C | A | non synonymous coding |
| 36 | Iron chelate uptake ABC transporter family permease subunit | ACL_RS04735 | 980639 | G | C | non synonymous coding |
| 37 | Antibiotic biosynthesis monooxygenase | ACL_RS04745 | 982687 | G | T | non synonymous coding |
| 38 | GGDEF domain-containing protein | ACL_RS06760 | 1429735 | G | A | stop gained |

^1^Name of the protein encoded by the mutant gene/functional category according to EggNOG; ^2^Locus of the gene on the chromosome *A.laidlawii* according to GenBank; ^3^SNP position in the nucleotide sequence of *A. laidlawii* PG8r1 strain; ^4^Nucleotide in *A. laidlawii* PG8Bc-3 strain; ^5^Nucleotide in *A. laidlawii* PG8r1 strain.
