# Supplementary material for: Data on the genome and proteome profiles of ciprofloxacin-resistant Acholeplasma laidlawii strains selected under different conditions in vitro
Source: Data Brief. 2020 Oct 19;33:106412. doi: 10.1016/j.dib.2020.106412 (PMC7585042; doi:10.1016/j.dib.2020.106412)
Supplement: Supplementary file 4 [file mmc4.docx]

**Supplementary table 4.** SNPs in genome of *A. laidlawii* PG8R_10_c-2

| № | Protein Name^1^ | Locus tag^2^ | Position^3^ | PG8B^4^ | PG8R_10_^5^ | Effect |
| --- | --- | --- | --- | --- | --- | --- |
|  | **Energy production and conversion** |  |  |  |  |  |
| 1 | Formate C-acetyltransferase | ACL_RS00145 | 32551 | G | T | non synonymous coding |
| 2 | Formate C-acetyltransferase | ACL_RS00145 | 33627 | T | G | non synonymous coding |
| 3 | RnfABCDGE type electron transport complex subunit C | ACL_RS00245 | 58432 | G | T | non synonymous coding |
| 4 | FAD-binding oxidoreductase | ACL_RS02115 | 443196 | G | T | non synonymous coding |
| 5 | LLM class flavin-dependent oxidoreductase | ACL_RS02645 | 558485 | T | G | stop gained |
| 6 | Nitroreductase family protein | ACL_RS03535 | 741899 | A | C | synonymous coding |
| 7 | Hypothetical protein | ACL_RS04840 | 1002284 | G | T | non synonymous coding |
| 8 | RnfABCDGE type electron transport complex subunit D | ACL_RS04780 | 990817 | T | G | non synonymous coding |
| 9 | Hypothetical protein | ACL_RS04840 | 1003394 | A | C | non synonymous coding |
| 10 | Aldehyde dehydrogenase family protein | ACL_RS06600 | 1392103 | C | A | non synonymous coding |
| 11 | SUF system NifU family Fe-S cluster assembly protein | ACL_RS06010 | 1262904 | C | A | non synonymous coding |
| 12 | Aldehyde dehydrogenase family protein | ACL_RS06600 | 1392783 | C | A | non synonymous coding |
| 13 | Type 2 isopentenyl-diphosphate Delta-isomerase | ACL_RS03955 | 834103 | T | G | non synonymous coding |
| 14 | Hypothetical protein | ACL_RS04840 | 1003758 | A | C | synonymous coding |
| 15 | F-type H+-transporting ATPase subunit epsilon | ACL_RS04850 | 1005071 | C | A | non synonymous coding |
| 16 | ATP synthase F0 subunit B | ACL_RS04875 | 1009621 | T | A | non synonymous coding |
| 17 | Aldo/keto reductase | ACL_RS04940 | 1021023 | C | A | non synonymous coding |
| 18 | V-type H+-transporting ATPase subunit I | ACL_RS05805 | 1227501 | G | T | non synonymous coding |
| 19 | V-type ATPase subunit | ACL_RS05810 | 1227784 | T | G | synonymous coding |
|  | **Cell cycle control, cell division, chromosome partitioning** |  |  |  |  |  |
| 20 | TRNA uridine-5-carboxymethylaminomethyl(34) synthesis enzyme MnmG | ACL_RS00315 | 71560 | T | G | non synonymous coding |
| 21 | Cell division protein FtsZ | ACL_RS03515 | 738882 | A | C | non synonymous coding |
|  | **Amino acid transport and metabolism** |  |  |  |  |  |
| 22 | Aminotransferase class I/II-fold pyridoxal phosphate-dependent enzyme | ACL_RS00115 | 28081 | G | T | non synonymous coding |
| 23 | 3-deoxy-7-phosphoheptulonate synthase | ACL_RS01060 | 207768 | G | T | non synonymous coding |
| 24 | Aminomethyl-transferring glycine dehydrogenase subunit GcvPB | ACL_RS01975 | 415634 | G | T | non synonymous coding |
| 25 | 3-deoxy-7-phosphoheptulonate synthase | ACL_RS01060 | 207943 | A | C | non synonymous coding |
| 26 | 3-dehydroquinate synthase | ACL_RS01070 | 210204 | T | G | non synonymous coding |
| 27 | Aminotransferase class I/II-fold pyridoxal phosphate-dependent enzyme | ACL_RS05195 | 1079985 | C | A | non synonymous coding |
| 28 | Shikimate kinase | ACL_RS04350 | 909545 | C | A | non synonymous coding |
| 29 | ABC transporter permease | ACL_RS02360 | 496718 | A | C | non synonymous coding |
| 30 | ABC transporter ATP-binding protein | ACL_RS02500 | 522915 | C | A | synonymous coding |
| 31 | Hypothetical protein | ACL_RS02820 | 599607 | A | C | non synonymous coding |
| 32 | ABC transporter ATP-binding protein | ACL_RS02505 | 523856 | G | T | stop gained |
| 33 | Transporter substrate-binding domain-containing protein | ACL_RS06195 | 1301792 | C | A | stop gained |
| 34 | Tryptophan synthase subunit beta | ACL_RS03860 | 813913 | A | C | non synonymous coding |
| 35 | Aspartate-semialdehyde dehydrogenase | ACL_RS03910 | 823681 | A | C | non synonymous coding |
| 36 | Diaminopimelate decarboxylase | ACL_RS03920 | 826080 | C | A | non synonymous coding |
| 37 | Diaminopimelate decarboxylase | ACL_RS03920 | 826555 | A | C | non synonymous coding |
| 38 | M42 family peptidase | ACL_RS04145 | 871589 | C | A | non synonymous coding |
| 39 | Carboxypeptidase M32 | ACL_RS04755 | 984725 | C | A | non synonymous coding |
| 40 | Carboxypeptidase M32 | ACL_RS04755 | 985055 | C | A | non synonymous coding |
| 41 | Carboxypeptidase M32 | ACL_RS04755 | 985118 | A | C | non synonymous coding |
| 42 | M3 family oligoendopeptidase | ACL_RS05050 | 1045077 | T | G | synonymous coding |
| 43 | ATP-binding cassette domain-containing protein | ACL_RS06035 | 1268468 | T | G | pseudo |
| 44 | ABC transporter ATP-binding protein | ACL_RS06040 | 1268971 | T | A | non synonymous coding |
| 45 | ABC transporter permease | ACL_RS07330 | 1269950 | C | A | non synonymous coding |
| 46 | ABC transporter permease | ACL_RS07330 | 1270592 | G | T | non synonymous coding |
| 47 | ABC transporter substrate-binding protein | ACL_RS06055 | 1273155 | C | T | synonymous coding |
| 48 | Amino acid ABC transporter ATP-binding protein | ACL_RS06190 | 1300960 | C | A | stop gained |
| 49 | Amino acid ABC transporter ATP-binding protein | ACL_RS06190 | 1301116 | A | C | non synonymous coding |
| 50 | Amino acid ABC transporter ATP-binding protein | ACL_RS06190 | 1301323 | C | A | non synonymous coding |
| 51 | ABC transporter substrate-binding protein | ACL_RS06365 | 1344969 | A | C | non synonymous coding |
| 52 | Agmatinase | ACL_RS06515 | 1374035 | A | C | non synonymous coding |
|  | **Nucleotide transport and metabolism** |  |  |  |  |  |
| 53 | Ribose-phosphate pyrophosphokinase | ACL_RS00095 | 20992 | T | G | non synonymous coding |
| 54 | Ribose-phosphate pyrophosphokinase | ACL_RS00095 | 21669 | C | A | non synonymous coding |
| 55 | Ribose-phosphate pyrophosphokinase | ACL_RS00095 | 21869 | G | T | non synonymous coding |
| 56 | Uracil phosphoribosyltransferase | ACL_RS01010 | 197530 | C | T | synonymous coding |
| 57 | Adenine phosphoribosyltransferase | ACL_RS04100 | 864021 | C | A | non synonymous coding |
| 58 | Anaerobic ribonucleoside triphosphate reductase | ACL_RS06400 | 1351141 | C | A | non synonymous coding |
| 59 | Guanylate kinase | ACL_RS01520 | 314078 | A | C | non synonymous coding |
| 60 | Deoxyribose-phosphate aldolase | ACL_RS03480 | 730683 | G | T | synonymous coding |
| 61 | Deoxyribose-phosphate aldolase | ACL_RS03480 | 730999 | T | G | non synonymous coding |
| 62 | Bifunctional metallophosphatase/5'-nucleotidase | ACL_RS03925 | 828039 | T | G | stop gained |
| 63 | DCMP deaminase family protein | ACL_RS04060 | 855558 | G | T | non synonymous coding |
| 64 | UMP kinase | ACL_RS05715 | 1211352 | G | T | synonymous coding |
| 65 | UMP kinase | ACL_RS05715 | 1211562 | A | C | non synonymous coding |
|  | **Carbohydrate transport and metabolism** |  |  |  |  |  |
| 66 | Extracellular solute-binding protein | ACL_RS07160 | 278233 | T | G | non synonymous coding |
| 67 | Extracellular solute-binding protein | ACL_RS01335 | 278811 | A | C | non synonymous coding |
| 68 | 4-alpha-glucanotransferase | ACL_RS03270 | 681620 | C | A | non synonymous coding |
| 69 | 6-phospho-beta-glucosidase | ACL_RS01300 | 264784 | G | T | non synonymous coding |
| 70 | Decarboxylating 6-phosphogluconate dehydrogenase | ACL_RS04765 | 987001 | A | C | non synonymous coding |
| 71 | Glycogen synthase | ACL_RS02600 | 542836 | G | T | non synonymous coding |
| 72 | Glycogen synthase | ACL_RS02600 | 542957 | C | A | synonymous coding |
| 73 | Hypothetical protein | ACL_RS06345 | 1339788 | C | A | non synonymous coding |
| 74 | Phosphoenolpyruvate-protein phosphotransferase | ACL_RS02185 | 458127 | C | A | non synonymous coding |
| 75 | Alpha-amylase | ACL_RS03330 | 700030 | T | G | non synonymous coding |
| 76 | Alpha-amylase | ACL_RS03330 | 700373 | G | T | non synonymous coding |
| 77 | Phosphocarrier protein HPr | ACL_RS01870 | 392952 | C | A | non synonymous coding |
| 78 | Glycoside hydrolase family 16 protein | ACL_RS03620 | 769945 | G | T | non synonymous coding |
| 79 | Extracellular solute-binding protein | ACL_RS03605 | 763897 | C | A | stop gained |
| 80 | Cellobiose phosphorylase | ACL_RS03630 | 771109 | G | T | non synonymous coding |
| 81 | Type I glyceraldehyde-3-phosphate dehydrogenase | ACL_RS05820 | 1229569 | G | A | synonymous coding |
| 82 | Phosphopyruvate hydratase | ACL_RS02010 | 422969 | C | A | non synonymous coding |
| 83 | Glucosamine-6-phosphate deaminase | ACL_RS01405 | 296549 | A | C | non synonymous coding |
| 84 | Alpha-glucosidase | ACL_RS03275 | 683390 | A | C | stop gained |
| 85 | Alpha-glucosidase | ACL_RS03275 | 683420 | C | A | non synonymous coding |
| 86 | Sugar ABC transporter permease | ACL_RS05280 | 1101678 | G | T | non synonymous coding |
| 87 | Carbohydrate ABC transporter permease | ACL_RS01370 | 289394 | C | A | non synonymous coding |
| 88 | Carbohydrate ABC transporter substrate-binding protein | ACL_RS00190 | 42044 | C | A | non synonymous coding |
| 89 | Cellobiose phosphorylase | ACL_RS03630 | 771406 | A | C | non synonymous coding |
| 90 | Phosphoglucomutase/phosphomannomutase family protein | ACL_RS05255 | 1093001 | T | G | non synonymous coding |
| 91 | Extracellular solute-binding protein | ACL_RS06975 | 1472472 | T | G | non synonymous coding |
| 92 | Sugar ABC transporter permease | ACL_RS06985 | 1474804 | A | G | non synonymous coding |
|  | **Coenzyme transport and metabolism** |  |  |  |  |  |
| 93 | Biotin-[acetyl-CoA-carboxylase] ligase | ACL_RS07200 | 491236 | T | G | non synonymous coding |
| 94 | GTP cyclohydrolase II | ACL_RS06540 | 1379782 | G | T | non synonymous coding |
| 95 | Bifunctional folylpolyglutamate synthase/dihydrofolate synthase | ACL_RS02720 | 579246 | C | A | non synonymous coding |
| 96 | Methionine adenosyltransferase | ACL_RS02990 | 631968 | A | C | synonymous coding |
| 97 | Bifunctional diaminohydroxyphosphoribosylaminopyrimidine deaminase/5-amino-6-(5-phosphoribosylamino)uracil reductase RibD | ACL_RS05470 | 1143219 | T | G | non synonymous coding |
|  | **Lipid transport and metabolism** |  |  |  |  |  |
| 98 | Cardiolipin synthase | ACL_RS00130 | 30323 | C | A | non synonymous coding |
| 99 | ACP S-malonyltransferase | ACL_RS02310 | 488191 | T | G | non synonymous coding |
| 100 | Sphingosine kinase | ACL_RS05860 | 1238530 | G | T | synonymous coding |
| 101 | Mevalonate kinase | ACL_RS03970 | 837543 | T | G | non synonymous coding |
| 102 | Acetyl-CoA carboxylase carboxyltransferase subunit beta | ACL_RS02295 | 485346 | T | G | non synonymous coding |
| 103 | Alpha/beta hydrolase | ACL_RS02725 | 579685 | G | T | non synonymous coding |
| 104 | 3-oxoacyl-ACP synthase | ACL_RS02530 | 529849 | T | G | non synonymous coding |
| 105 | Acyl-ACP thioesterase | ACL_RS03940 | 832013 | A | C | non synonymous coding |
| 106 | Acyl-CoA dehydrogenase family protein | ACL_RS05485 | 1146693 | A | G | non synonymous coding |
| 107 | DUF92 domain-containing protein | ACL_RS06995 | 1477719 | G | T | non synonymous coding |
|  | **Inorganic ion transport and metabolism** |  |  |  |  |  |
| 108 | Sugar ABC transporter permease | ACL_RS07240 | 687746 | G | T | non synonymous coding |
| 109 | ABC transporter ATP-binding protein | ACL_RS03850 | 811259 | T | G | synonymous coding |
| 110 | Superoxide dismutase | ACL_RS01510 | 311962 | C | T | non synonymous coding |
| 111 | ABC transporter ATP-binding protein | ACL_RS03850 | 811273 | C | T | non synonymous coding |
| 112 | ABC transporter ATP-binding protein | ACL_RS03850 | 811489 | A | C | non synonymous coding |
| 113 | ABC transporter substrate-binding protein | ACL_RS04960 | 1024740 | A | C | synonymous coding |
| 114 | ATP-binding cassette domain-containing protein | ACL_RS04730 | 979898 | C | A | non synonymous coding |
| 115 | Iron chelate uptake ABC transporter family permease subunit | ACL_RS04740 | 981728 | A | C | non synonymous coding |
| 116 | Cadmium-translocating P-type ATPase | ACL_RS06230 | 1315116 | T | G | non synonymous coding |
| 117 | Magnesium-translocating P-type ATPase | ACL_RS06340 | 1337068 | G | T | non synonymous coding |
| 118 | Zinc ABC transporter substrate-binding protein | ACL_RS05440 | 1138290 | A | C | stop gained |
| 119 | ABC transporter substrate-binding protein | ACL_RS04960 | 1025288 | C | A | non synonymous coding |
| 120 | ABC transporter permease | ACL_RS06355 | 1342523 | T | G | non synonymous coding |
| 121 | ABC transporter permease | ACL_RS06355 | 1342703 | A | G | non synonymous coding |
|  | **Secondary metabolites biosynthesis, transport, and catabolism** |  |  |  |  |  |
| 122 | Pseudouridine-5'-phosphate glycosidase | ACL_RS03980 | 838951 | T | G | non synonymous coding |
|  | Defense mechanisms |  |  |  |  |  |
| 123 | DUF1343 domain-containing protein | ACL_RS01380 | 290938 | G | T | synonymous coding |
| 124 | ABC transporter ATP-binding protein/permease | ACL_RS07180 | 357894 | T | G | non synonymous coding |
| 125 | ABC transporter ATP-binding protein/permease | ACL_RS07180 | 358865 | G | T | non synonymous coding |
| 126 | ABC transporter ATP-binding protein | ACL_RS07265 | 745856 | C | A | stop gained |
| 127 | ABC transporter ATP-binding protein | ACL_RS04180 | 878248 | T | G | non synonymous coding |
| 128 | ABC transporter ATP-binding protein | ACL_RS01140 | 224327 | G | T | non synonymous coding |
| 129 | ABC transporter ATP-binding protein | ACL_RS04180 | 879430 | A | G | non synonymous coding |
| 130 | ABC transporter permease | ACL_RS07020 | 1482190 | G | T | non synonymous coding |
| 131 | ABC transporter ATP-binding protein | ACL_RS01145 | 227281 | C | A | non synonymous coding |
| 132 | ABC transporter ATP-binding protein | ACL_RS04655 | 965931 | T | C | non synonymous coding |
| 133 | ABC transporter permease | ACL_RS07020 | 1482596 | C | A | non synonymous coding |
| 134 | ABC transporter ATP-binding protein | ACL_RS04390 | 921555 | C | A | non synonymous coding |
| 135 | ABC transporter ATP-binding protein | ACL_RS04915 | 1017215 | C | A | non synonymous coding |
| 136 | Hypothetical protein | ACL_RS04990 | 1033264 | T | A | non synonymous coding |
| 137 | ABC transporter ATP-binding protein | ACL_RS06125 | 1287920 | C | A | non synonymous coding |
| 138 | ABC transporter ATP-binding protein | ACL_RS02095 | 438185 | A | C | stop gained |
| 139 | ABC transporter ATP-binding protein | ACL_RS06125 | 1288120 | G | T | non synonymous coding |
| 140 | Type I restriction endonuclease subunit R | ACL_RS06280 | 1327286 | C | A | stop gained |
| 141 | SAM-dependent DNA methyltransferase | ACL_RS06295 | 1330894 | C | A | non synonymous coding |
|  | **Replication, recombination and repair** |  |  |  |  |  |
| 142 | DNA gyrase subunit A | ACL_RS00040 | 7879 | G | T | synonymous coding |
| 143 | DNA gyrase subunit A | ACL_RS00040 | 7925 | G | T | non synonymous coding |
| 144 | DNA gyrase subunit A | ACL_RS00040 | 9002 | G | T | non synonymous coding |
| 145 | Transcription-repair coupling factor | ACL_RS00105 | 23012 | A | C | synonymous coding |
| 146 | Endonuclease | ACL_RS07130 | 138207 | A | C | synonymous coding |
| 147 | Endonuclease | ACL_RS07130 | 139754 | C | A | non synonymous coding |
| 148 | 8-oxo-dGTP diphosphatase | ACL_RS04230 | 887911 | T | G | non synonymous coding |
| 149 | 8-oxo-dGTP diphosphatase | ACL_RS06160 | 1296469 | G | T | non synonymous coding |
| 150 | Recombinase family protein | ACL_RS02900 | 614803 | T | G | stop gained |
| 151 | Recombination protein RecR | ACL_RS06925 | 1460909 | G | C | synonymous coding |
| 152 | Hypothetical protein | ACL_RS02075 | 434762 | G | T | non synonymous coding |
| 153 | Replication-associated recombination protein A | ACL_RS04105 | 864750 | C | A | non synonymous coding |
| 154 | PolC-type DNA polymerase III | ACL_RS01215 | 244573 | T | G | non synonymous coding |
| 155 | Ribonuclease HII | ACL_RS01690 | 350318 | T | G | non synonymous coding |
| 156 | DnaD domain protein | ACL_RS01760 | 370640 | T | C | non synonymous coding |
| 157 | Excinuclease ABC subunit UvrA | ACL_RS06665 | 1406982 | G | T | non synonymous coding |
| 158 | Excinuclease ABC subunit UvrC | ACL_RS01530 | 314956 | C | A | non synonymous coding |
| 159 | DNA repair protein RadC | ACL_RS01800 | 379874 | C | A | non synonymous coding |
| 160 | Holliday junction branch migration DNA helicase RuvB | ACL_RS01850 | 389845 | G | T | non synonymous coding |
| 161 | DNA topoisomerase IV subunit B | ACL_RS01895 | 397721 | T | G | non synonymous coding |
| 162 | DNA topoisomerase IV subunit B | ACL_RS01895 | 397959 | C | A | non synonymous coding |
| 163 | DNA topoisomerase IV subunit A | ACL_RS01900 | 398859 | C | T | non synonymous coding |
| 164 | DNA repair protein RecN | ACL_RS02395 | 502360 | G | T | non synonymous coding |
| 165 | DUF4011 domain-containing protein | ACL_RS02620 | 555043 | T | G | non synonymous coding |
| 166 | Site-specific tyrosine recombinase XerD | ACL_RS03380 | 713724 | C | A | synonymous coding |
| 167 | Endonuclease MutS2 | ACL_RS04030 | 849417 | C | A | non synonymous coding |
| 168 | Endonuclease MutS2 | ACL_RS04030 | 849693 | A | C | non synonymous coding |
| 169 | Endonuclease MutS2 | ACL_RS04030 | 850739 | C | T | non synonymous coding |
| 170 | DNA mismatch repair endonuclease MutL | ACL_RS04360 | 911557 | A | C | non synonymous coding |
| 171 | DNA mismatch repair protein MutS | ACL_RS04365 | 912312 | A | C | non synonymous coding |
| 172 | DNA mismatch repair protein MutS | ACL_RS04365 | 914498 | C | A | non synonymous coding |
| 173 | UvrD-helicase domain-containing protein | ACL_RS05290 | 1106270 | G | T | non synonymous coding |
| 174 | Deoxyribodipyrimidine photo-lyase | ACL_RS07055 | 1489152 | G | T | synonymous coding |
|  | **Transcription** |  |  |  |  |  |
| 175 | LacI family DNA-binding transcriptional regulator | ACL_RS00185 | 41824 | T | G | non synonymous coding |
| 176 | DNA-directed RNA polymerase subunit beta | ACL_RS00840 | 154955 | C | A | non synonymous coding |
| 177 | Crp/Fnr family transcriptional regulator | ACL_RS05490 | 1147058 | A | G | non synonymous coding |
| 178 | Helix-turn-helix transcriptional regulator | ACL_RS07195 | 466967 | C | A | non synonymous coding |
| 179 | Transcriptional regulator | ACL_RS04235 | 889232 | T | G | non synonymous coding |
| 180 | Helix-turn-helix transcriptional regulator | ACL_RS07345 | 1335419 | A | C | non synonymous coding |
| 181 | Helix-turn-helix transcriptional regulator | ACL_RS07345 | 1335667 | C | A | non synonymous coding |
| 182 | Phage antirepressor KilAC domain-containing protein | ACL_RS02920 | 619172 | G | T | non synonymous coding |
| 183 | DNA-directed RNA polymerase subunit beta' | ACL_RS00845 | 159557 | G | T | non synonymous coding |
| 184 | Ribosome biogenesis GTPase YlqF | ACL_RS01685 | 349453 | T | G | non synonymous coding |
| 185 | DNA-directed RNA polymerase subunit beta' | ACL_RS00845 | 159994 | C | A | non synonymous coding |
| 186 | Ribonuclease III family protein | ACL_RS03180 | 663324 | G | T | non synonymous coding |
| 187 | Transcription termination/antitermination protein NusA | ACL_RS01585 | 327625 | A | C | non synonymous coding |
| 188 | Helix-turn-helix transcriptional regulator | ACL_RS02905 | 617069 | G | T | synonymous coding |
| 189 | Bifunctional (p)ppGpp synthetase/guanosine-3',5'-bis(diphosphate) 3'-pyrophosphohydrolase | ACL_RS04095 | 862564 | G | T | non synonymous coding |
|  | **Translation, ribosomal structure and biogenesis** |  |  |  |  |  |
| 190 | 50S ribosomal protein L3 | ACL_RS00430 | 84462 | A | C | non synonymous coding |
| 191 | 50S ribosomal protein L13 | ACL_RS00680 | 120297 | C | A | non synonymous coding |
| 192 | 50S ribosomal protein L29 | ACL_RS00470 | 88619 | G | T | non synonymous coding |
| 193 | 30S ribosomal protein S1 | ACL_RS04325 | 906235 | A | C | non synonymous coding |
| 194 | 30S ribosomal protein S7 | ACL_RS00910 | 178760 | G | T | synonymous coding |
| 195 | 50S ribosomal protein L14 | ACL_RS00480 | 89296 | C | A | non synonymous coding |
| 196 | Elongation factor G | ACL_RS00915 | 179880 | C | A | non synonymous coding |
| 197 | Elongation factor G | ACL_RS01490 | 308095 | T | G | stop gained |
| 198 | 23S rRNA (uracil(1939)-C(5))-methyltransferase RlmD | ACL_RS02550 | 533232 | C | A | synonymous coding |
| 199 | 23S rRNA (uracil(1939)-C(5))-methyltransferase RlmD | ACL_RS02570 | 537360 | C | A | synonymous coding |
| 200 | Alanine-tRNA ligase | ACL_RS04480 | 936044 | G | A | non synonymous coding |
| 201 | Alanine-tRNA ligase | ACL_RS04480 | 936410 | C | A | non synonymous coding |
| 202 | RluA family pseudouridine synthase | ACL_RS04040 | 852734 | C | A | non synonymous coding |
| 203 | Glutamate-tRNA ligase | ACL_RS00690 | 121351 | G | T | non synonymous coding |
| 204 | Threonine-tRNA ligase | ACL_RS01770 | 373104 | C | A | synonymous coding |
| 205 | Translation initiation factor IF-2 | ACL_RS01600 | 329572 | C | A | non synonymous coding |
| 206 | 16S rRNA processing protein RimM | ACL_RS05885 | 1243091 | T | G | non synonymous coding |
| 207 | Threonine-tRNA ligase | ACL_RS01770 | 373834 | G | T | non synonymous coding |
| 208 | Isoleucine-tRNA ligase | ACL_RS03495 | 733733 | G | T | non synonymous coding |
| 209 | TRNA pseudouridine(55) synthase TruB | ACL_RS04020 | 848182 | C | A | non synonymous coding |
| 210 | TRNA pseudouridine(55) synthase TruB | ACL_RS04020 | 848214 | G | T | non synonymous coding |
| 211 | S1 RNA-binding domain-containing protein | ACL_RS06725 | 1425072 | A | C | start lost |
| 212 | Bifunctional oligoribonuclease/PAP phosphatase NrnA | ACL_RS06955 | 1466112 | A | C | synonymous coding |
| 213 | Bifunctional oligoribonuclease/PAP phosphatase NrnA | ACL_RS06955 | 1466475 | A | C | non synonymous coding |
|  | **Signal transduction mechanisms** |  |  |  |  |  |
| 214 | GGDEF domain-containing protein | ACL_RS00855 | 163445 | A | C | non synonymous coding |
| 215 | Arsenate reductase ArsC | ACL_RS02455 | 513281 | A | C | non synonymous coding |
| 216 | HPr(Ser) kinase/phosphatase | ACL_RS06655 | 1403792 | G | T | non synonymous coding |
|  | **Post-translational modification, protein turnover, and chaperones** |  |  |  |  |  |
| 217 | Ribonuclease J | ACL_RS01545 | 319609 | A | C | non synonymous coding |
| 218 | Insulinase family protein | ACL_RS03470 | 729065 | A | C | stop gained |
| 219 | Ribonuclease J | ACL_RS04115 | 866953 | C | A | non synonymous coding |
| 220 | Hypothetical protein | ACL_RS00800 | 148959 | G | T | non synonymous coding |
| 221 | Thioredoxin | ACL_RS04025 | 849109 | G | A | synonymous coding |
| 222 | Hsp33 family molecular chaperone HslO | ACL_RS06820 | 1442516 | A | C | non synonymous coding |
| 223 | Molecular chaperone DnaJ | ACL_RS02765 | 587702 | A | C | synonymous coding |
| 224 | AAA family ATPase | ACL_RS05560 | 1187926 | C | A | stop gained |
| 225 | FAD-dependent oxidoreductase | ACL_RS05960 | 1255245 | T | G | non synonymous coding |
| 226 | Chaperonin GroEL | ACL_RS06175 | 1297697 | A | C | non synonymous coding |
| 227 | FAD-dependent oxidoreductase | ACL_RS06645 | 1401654 | A | C | non synonymous coding |
| 228 | ATP-dependent zinc metalloprotease FtsH | ACL_RS06855 | 1448527 | T | G | synonymous coding |
|  | **Cell wall/membrane/envelope biogenesis** |  |  |  |  |  |
| 229 | UDP-N-acetylglucosamine 2-epimerase (non-hydrolyzing) | ACL_RS05140 | 1068726 | G | T | non synonymous coding |
|  | **Intracellular trafficking, secretion, and vesicular transport** |  |  |  |  |  |
| 230 | Signal recognition particle protein | ACL_RS01745 | 364532 | C | A | non synonymous coding |
|  | **Function unknown** |  |  |  |  |  |
| 231 | HD domain-containing protein | ACL_RS00075 | 18548 | G | C | non synonymous coding |
| 232 | YitT family protein | ACL_RS00660 | 116757 | T | G | non synonymous coding |
| 233 | YitT family protein | ACL_RS00660 | 117360 | C | A | non synonymous coding |
| 234 | Family 10 glycosylhydrolase | ACL_RS00895 | 175776 | G | T | non synonymous coding |
| 235 | Family 10 glycosylhydrolase | ACL_RS00895 | 175815 | T | G | non synonymous coding |
| 236 | CinA family protein | ACL_RS07135 | 185485 | T | G | non synonymous coding |
| 237 | MBL fold metallo-hydrolase | ACL_RS01040 | 204397 | A | C | non synonymous coding |
| 238 | DegV family protein | ACL_RS01055 | 206396 | G | T | non synonymous coding |
| 239 | MgtC/SapB family protein | ACL_RS06945 | 1464689 | G | T | non synonymous coding |
| 240 | Holliday junction resolvase RecU | ACL_RS04120 | 868070 | C | A | non synonymous coding |
| 241 | RRNA maturation RNase YbeY | ACL_RS04700 | 975831 | G | T | non synonymous coding |
| 242 | Beta-propeller domain-containing protein | ACL_RS01610 | 332419 | C | A | stop gained |
| 243 | DUF368 domain-containing protein | ACL_RS01875 | 393610 | A | C | synonymous coding |
| 244 | YaaA family protein | ACL_RS00160 | 36724 | T | G | non synonymous coding |
| 245 | 1-acyl-sn-glycerol-3-phosphate acyltransferase | ACL_RS03195 | 667449 | T | G | stop gained |
| 246 | AAA family ATPase | ACL_RS02180 | 456045 | C | A | non synonymous coding |
| 247 | DegV family protein | ACL_RS02700 | 574002 | G | T | non synonymous coding |
| 248 | Phage major capsid protein | ACL_RS03055 | 643471 | A | C | synonymous coding |
| 249 | Phage major capsid protein | ACL_RS03055 | 643934 | G | T | non synonymous coding |
| 250 | Hypothetical protein | ACL_RS03090 | 647457 | T | C | synonymous coding |
| 251 | Hypothetical protein | ACL_RS03100 | 650182 | C | A | synonymous coding |
| 252 | Hypothetical protein | ACL_RS03305 | 693149 | C | A | stop gained |
| 253 | Hypothetical protein | ACL_RS03565 | 751283 | A | C | non synonymous coding |
| 254 | Hypothetical protein | ACL_RS03585 | 756736 | G | T | synonymous coding |
| 255 | Hypothetical protein | ACL_RS03585 | 757351 | A | C | non synonymous coding |
| 256 | Hypothetical protein | ACL_RS03585 | 757409 | T | G | non synonymous coding |
| 257 | Hypothetical protein | ACL_RS03585 | 758329 | T | G | synonymous coding |
| 258 | YIP1 family protein | ACL_RS03590 | 759750 | G | T | non synonymous coding |
| 259 | YIP1 family protein | ACL_RS03590 | 760185 | A | G | non synonymous coding |
| 260 | Hypothetical protein | ACL_RS04130 | 869671 | A | C | synonymous coding |
| 261 | Ribonuclease Y | ACL_RS04380 | 918840 | C | T | non synonymous coding |
| 262 | 50S ribosome-binding GTPase | ACL_RS04445 | 931682 | C | A | non synonymous coding |
| 263 | 50S ribosome-binding GTPase | ACL_RS04445 | 931945 | A | C | non synonymous coding |
| 264 | SPFH domain-containing protein | ACL_RS04600 | 958787 | G | T | synonymous coding |
| 265 | SPFH domain-containing protein | ACL_RS04600 | 958818 | C | A | non synonymous coding |
| 266 | SAM-dependent methyltransferase | ACL_RS04665 | 968923 | G | T | non synonymous coding |
| 267 | 16S rRNA (uracil(1498)-N(3))-methyltransferase | ACL_RS04715 | 977593 | G | T | non synonymous coding |
| 268 | NYN domain-containing protein | ACL_RS05025 | 1041622 | C | A | non synonymous coding |
| 269 | Aminoacetone oxidase family FAD-binding enzyme | ACL_RS05065 | 1050253 | C | A | non synonymous coding |
| 270 | YwaF family protein | ACL_RS05080 | 1052680 | T | G | non synonymous coding |
| 271 | DUF853 family protein | ACL_RS05215 | 1084559 | T | G | non synonymous coding |
| 272 | Cation:proton antiporter | ACL_RS03415 | 721439 | A | C | stop gained |
| 273 | TRNA (guanosine(46)-N7)-methyltransferase TrmB | ACL_RS01880 | 394249 | C | A | non synonymous coding |
| 274 | HAD-IA family hydrolase | ACL_RS04555 | 951722 | T | C | non synonymous coding |
| 275 | FMN-binding protein | ACL_RS07300 | 992811 | A | C | non synonymous coding |
| 276 | Cation:proton antiporter | ACL_RS03415 | 720684 | C | A | synonymous coding |
| 277 | InlB B-repeat-containing protein | ACL_RS05335 | 1113303 | T | G | stop gained |
| 278 | CidA/LrgA family protein | ACL_RS05465 | 1142477 | G | T | non synonymous coding |
| 279 | Putative sulfate exporter family transporter | ACL_RS05505 | 1149680 | G | T | non synonymous coding |
| 280 | Na+/glutamate symporter | ACL_RS03200 | 668254 | T | G | stop gained |
| 281 | C39 family peptidase | ACL_RS05510 | 1150643 | T | G | non synonymous coding |
| 282 | AI-2E family transporter | ACL_RS04085 | 860402 | T | G | non synonymous coding |
| 283 | InlB B-repeat-containing protein | ACL_RS05535 | 1166947 | C | A | non synonymous coding |
| 284 | InlB B-repeat-containing protein | ACL_RS05535 | 1168737 | G | T | non synonymous coding |
| 285 | InlB B-repeat-containing protein | ACL_RS05535 | 1178659 | T | G | non synonymous coding |
| 286 | InlB B-repeat-containing protein | ACL_RS05535 | 1180506 | A | C | non synonymous coding |
| 287 | InlB B-repeat-containing protein | ACL_RS06065 | 1275425 | C | A | non synonymous coding |
| 288 | InlB B-repeat-containing protein | ACL_RS06065 | 1275698 | G | T | non synonymous coding |
| 289 | InlB B-repeat-containing protein | ACL_RS06065 | 1275832 | G | T | stop gained |
| 290 | InlB B-repeat-containing protein | ACL_RS06680 | 1413245 | G | T | non synonymous coding |
| 291 | Divergent PAP2 family protein | ACL_RS06740 | 1426836 | G | T | synonymous coding |
| 292 | NCS2 family permease | ACL_RS06780 | 1433173 | C | A | non synonymous coding |
| 293 | NCS2 family permease | ACL_RS06780 | 1433202 | G | T | non synonymous coding |
| 294 | DUF4097 family beta strand repeat protein | ACL_RS06825 | 1443033 | A | C | non synonymous coding |
| 295 | DegV family EDD domain-containing protein | ACL_RS07045 | 1487474 | C | A | non synonymous coding |
|  | **Not in EggNog** |  |  |  |  |  |
| 296 | N-acetylmuramoyl-L-alanine amidase | ACL_RS00215 | 49861 | C | A | non synonymous coding |
| 297 | N-acetylmuramoyl-L-alanine amidase | ACL_RS00215 | 50868 | G | T | non synonymous coding |
| 298 | Hypothetical protein | ACL_RS00255 | 60893 | C | A | non synonymous coding |
| 299 | Chloride channel protein | ACL_RS02225 | 465110 | G | T | stop gained |
| 300 | Hypothetical protein | ACL_RS05225 | 1086771 | C | A | stop gained |
| 301 | Hypothetical protein | ACL_RS05270 | 1100442 | C | A | stop gained |
| 302 | Family 10 glycosylhydrolase | ACL_RS07150 | 269261 | G | T | non synonymous coding |
| 303 | Hypothetical protein | ACL_RS01360 | 287665 | T | G | non synonymous coding |
| 304 | RecX family transcriptional regulator | ACL_RS01665 | 345174 | C | A | non synonymous coding |
| 305 | InlB B-repeat-containing protein | ACL_RS01700 | 354457 | T | G | non synonymous coding |
| 306 | Nucleoside kinase | ACL_RS01905 | 401382 | C | A | non synonymous coding |
| 307 | Nucleoside kinase | ACL_RS01905 | 401905 | G | T | non synonymous coding |
| 308 | Hypothetical protein | ACL_RS02020 | 424769 | G | T | non synonymous coding |
| 309 | Hypothetical protein | ACL_RS02735 | 582473 | G | T | non synonymous coding |
| 310 | Hypothetical protein | ACL_RS03105 | 651562 | G | T | non synonymous coding |
| 311 | Hypothetical protein | ACL_RS03110 | 652172 | G | T | non synonymous coding |
| 312 | Hypothetical protein | ACL_RS03175 | 662276 | C | A | non synonymous coding |
| 313 | ABC transporter permease | ACL_RS03770 | 796376 | T | G | non synonymous coding |
| 314 | Hypothetical protein | ACL_RS03820 | 805997 | C | A | non synonymous coding |
| 315 | Hypothetical protein | ACL_RS04595 | 957848 | C | A | non synonymous coding |
| 316 | Hypothetical protein | ACL_RS04985 | 1030987 | T | G | non synonymous coding |
| 317 | Hypothetical protein | ACL_RS05045 | 1044155 | G | T | non synonymous coding |
| 318 | Glycoside hydrolase family 16 protein | ACL_RS05260 | 1095392 | A | C | non synonymous coding |
| 319 | Ig-like domain-containing protein | ACL_RS06000 | 1260270 | G | T | non synonymous coding |
| 320 | Hypothetical protein | ACL_RS06565 | 1383871 | G | T | non synonymous coding |
| 321 | Hypothetical protein | ACL_RS06610 | 1395337 | G | T | non synonymous coding |
| 322 | Hypothetical protein | ACL_RS04370 | 916385 | T | G | synonymous coding |
| 323 | Leucine-rich repeat domain-containing protein | ACL_RS07350 | 1393024 | C | A | synonymous coding |
| 324 | HAD-IC family P-type ATPase | ACL_RS00720 | 129543 | T | G | non synonymous coding |
| 325 | HAD-IC family P-type ATPase | ACL_RS00720 | 130532 | C | T | non synonymous coding |
| 326 | HAD-IC family P-type ATPase | ACL_RS00720 | 131390 | G | T | non synonymous coding |
| 327 | Class I SAM-dependent methyltransferase | ACL_RS00835 | 154279 | A | G | stop gained |
| 328 | Trypsin-like peptidase domain-containing protein | ACL_RS00935 | 184968 | C | A | non synonymous coding |
| 329 | Hypothetical protein | ACL_RS01210 | 241926 | T | G | non synonymous coding |
| 330 | DNA translocase FtsK | ACL_RS01680 | 348860 | T | G | non synonymous coding |
| 331 | Hypothetical protein | ACL_RS01835 | 384744 | G | T | non synonymous coding |
| 332 | Hypothetical protein | ACL_RS01840 | 385903 | T | G | non synonymous coding |
| 333 | Hypothetical protein | ACL_RS01840 | 388664 | C | A | synonymous coding |
| 334 | Hypothetical protein | ACL_RS02210 | 463458 | G | T | non synonymous coding |
| 335 | Alpha-glucosidase C-terminal domain-containing protein | ACL_RS02245 | 470412 | C | A | non synonymous coding |
| 336 | Integral membrane protein | ACL_RS02345 | 493851 | A | C | non synonymous coding |
| 337 | IS3 family transposase | ACL_RS07390 | 606264 | C | A | non synonymous coding |
| 338 | AAA family ATPase | ACL_RS02870 | 609120 | A | C | non synonymous coding |
| 339 | AAA family ATPase | ACL_RS02870 | 610932 | G | T | stop gained |
| 340 | DUF4297 domain-containing protein | ACL_RS03000 | 633781 | G | T | stop gained |
| 341 | FtsX-like permease family protein | ACL_RS03215 | 671110 | G | T | non synonymous coding |
| 342 | FtsX-like permease family protein | ACL_RS03215 | 672624 | C | A | synonymous coding |
| 343 | ABC transporter ATP-binding protein | ACL_RS07290 | 968433 | A | C | non synonymous coding |
| 344 | ABC transporter ATP-binding protein | ACL_RS07290 | 968485 | C | A | non synonymous coding |
| 345 | Redox-regulated ATPase YchF | ACL_RS03510 | 737378 | G | T | non synonymous coding |
| 346 | M48 family metallopeptidase | ACL_RS07275 | 933556 | C | A | non synonymous coding |
| 347 | Type I pullulanase | ACL_RS03320 | 696434 | G | T | non synonymous coding |
| 348 | Type I pullulanase | ACL_RS03320 | 697532 | T | G | non synonymous coding |
| 349 | Hypothetical protein | ACL_RS03450 | 726622 | A | C | non synonymous coding |
| 350 | EAL domain-containing protein | ACL_RS04280 | 894986 | T | G | non synonymous coding |
| 351 | EAL domain-containing protein | ACL_RS04280 | 897556 | A | T | non synonymous coding |
| 352 | Hypothetical protein | ACL_RS04975 | 1029240 | A | C | non synonymous coding |
| 353 | Hypothetical protein | ACL_RS05000 | 1034173 | G | T | non synonymous coding |
| 354 | Hypothetical protein | ACL_RS05000 | 1034281 | G | T | non synonymous coding |
| 355 | Hypothetical protein | ACL_RS05000 | 1034839 | T | G | non synonymous coding |
| 356 | Hypothetical protein | ACL_RS05005 | 1035405 | T | G | non synonymous coding |
| 357 | Hypothetical protein | ACL_RS05005 | 1035531 | T | G | non synonymous coding |
| 358 | Hypothetical protein | ACL_RS05010 | 1036690 | G | T | stop gained |
| 359 | Transcriptional regulator | ACL_RS05095 | 1055509 | T | G | non synonymous coding |
| 360 | Hypothetical protein | ACL_RS05250 | 1090727 | T | G | non synonymous coding |
| 361 | Hypothetical protein | ACL_RS05250 | 1090829 | G | C | non synonymous coding |
| 362 | GNAT family N-acetyltransferase | ACL_RS05450 | 1139569 | G | T | non synonymous coding |
| 363 | Cadherin-like beta sandwich domain-containing protein | ACL_RS05530 | 1156899 | A | C | non synonymous coding |
| 364 | Bifunctional diguanylate cyclase/phosphodiesterase | ACL_RS05015 | 1037446 | T | G | non synonymous coding |
| 365 | GHKL domain-containing protein | ACL_RS07035 | 1485356 | T | G | non synonymous coding |
| 366 | DUF3899 domain-containing protein | ACL_RS06060 | 1274798 | T | G | non synonymous coding |
| 367 | DUF4373 domain-containing protein | ACL_RS07215 | 620791 | C | A | non synonymous coding |
| 368 | EAL domain-containing protein | ACL_RS05020 | 1040778 | T | G | non synonymous coding |
| 369 | HD domain-containing protein | ACL_RS04490 | 939655 | C | A | non synonymous coding |
| 370 | Cadherin-like beta sandwich domain-containing protein | ACL_RS05530 | 1162660 | A | C | non synonymous coding |
| 371 | SHOCT domain-containing protein | ACL_RS05570 | 1189976 | T | G | non synonymous coding |
| 372 | Folate family ECF transporter S component | ACL_RS07325 | 1218435 | C | A | stop gained |
| 373 | Hypothetical protein | ACL_RS05920 | 1247643 | A | C | stop gained |
| 374 | Alkaline phosphatase family protein | ACL_RS06095 | 1281698 | G | T | non synonymous coding |
| 375 | Elongation factor 4 | ACL_RS03370 | 709985 | C | A | synonymous coding |
| 376 | DNA polymerase III subunit beta | ACL_RS00020 | 3524 | G | T | non synonymous coding |
| 377 | Hypothetical protein | ACL_RS06165 | 1296961 | C | A | non synonymous coding |
| 378 | InlB B-repeat-containing protein | ACL_RS06275 | 1323593 | C | A | non synonymous coding |
| 379 | Integral membrane protein | ACL_RS06475 | 1366016 | A | T | non synonymous coding |
| 380 | Hypothetical protein | ACL_RS06880 | 1452045 | C | A | non synonymous coding |
| 381 | Hypothetical protein | ACL_RS07070 | 1493246 | T | G | synonymous coding |
| 382 | Sensor domain-containing diguanylate cyclase | ACL_RS07115 | 119892 | A | C | non synonymous coding |
| 383 | Peptide chain release factor N(5)-glutamine methyltransferase | ACL_RS00990 | 193878 | A | C | non synonymous coding |
| 384 | Hypothetical protein | ACL_RS01325 | 275401 | C | A | stop gained |
| 385 | McrC family protein | ACL_RS03165 | 660915 | T | G | non synonymous coding |
| 386 | Hypothetical protein | ACL_RS06965 | 1468886 | A | C | non synonymous coding |
| 387 | Hypothetical protein | ACL_RS06965 | 1469105 | C | A | non synonymous coding |
|  | **Pseudogenes** |  |  |  |  |  |
| 388 | ABC transporter ATP-binding protein (pseudo) | ACL_RS02090 | 436448 | C | A | pseudo |
| 389 | ABC transporter ATP-binding protein (pseudo) | ACL_RS02090 | 437243 | G | T | pseudo |
| 390 | ParB N-terminal domain-containing protein (pseudo) | ACL_RS02965 | 629222 | A | C | pseudo |
| 391 | ParB N-terminal domain-containing protein (pseudo) | ACL_RS02965 | 630092 | A | C | pseudo |
| 392 |  |  | 73398 | C | A | pseudo |
| 393 |  |  | 78207 | A | C | pseudo |
| 394 |  |  | 98557 | C | T | pseudo |
| 395 |  |  | 98640 | G | T | pseudo |
| 396 |  |  | 146754 | C | A | pseudo |
| 397 |  |  | 163095 | G | T | pseudo |
| 398 |  |  | 384522 | C | A | pseudo |
| 399 |  |  | 406001 | T | G | pseudo |
| 400 | ABC transporter ATP-binding protein (pseudo) | ACL_RS01940 | 408037 | G | T | pseudo |
| 401 |  |  | 450918 | T | G | pseudo |
| 402 |  |  | 526889 | T | G | pseudo |
| 403 |  |  | 556236 | C | A | pseudo |
| 404 |  |  | 583941 | G | T | pseudo |
| 405 |  |  | 583950 | T | G | pseudo |
| 406 |  |  | 614585 | C | A | pseudo |
| 407 |  |  | 656468 | G | T | pseudo |
| 408 |  |  | 802991 | C | A | pseudo |
| 409 |  |  | 809107 | C | A | pseudo |
| 410 |  |  | 872415 | T | G | pseudo |
| 411 |  |  | 922117 | A | G | pseudo |
| 412 |  |  | 950861 | G | T | pseudo |
| 413 |  |  | 1023237 | G | T | pseudo |
| 414 |  |  | 1035084 | C | A | pseudo |
| 415 |  |  | 1037115 | G | T | pseudo |
| 416 |  |  | 1057085 | C | A | pseudo |
| 417 |  |  | 1082475 | A | G | pseudo |
| 418 |  |  | 1111729 | G | T | pseudo |
| 419 |  |  | 1124140 | A | C | pseudo |
| 420 |  |  | 1205414 | T | G | pseudo |
| 421 |  |  | 1218996 | G | T | pseudo |
| 422 |  |  | 1291699 | T | G | pseudo |
| 423 |  |  | 1297600 | A | C | pseudo |
| 424 |  |  | 1312907 | G | T | pseudo |
| 425 |  |  | 1333365 | C | A | pseudo |
| 426 |  |  | 1388747 | G | T | pseudo |
| 427 | C4-dicarboxylate transporter DcuC (pseudo) | ACL_RS06785 | 1435174 | A | C | pseudo |
| 428 |  |  | 1460309 | C | A | pseudo |
| 429 |  |  | 1477260 | T | G | pseudo |

^1^The name of the protein encoded by the mutant gene/functional category according to EggNOG; ^2^Locus of the gene on the chromosome *A.laidlawii* according to GenBank; ^3^SNP position in the nucleotide sequence of *A. laidlawii* PG8R_10_c-2 strain; ^4^Nucleotide in *A. laidlawii* PG8Bc-3 strain; ^5^Nucleotide in *A. laidlawii* PG8R_10_c-2 strain.
