# Supplementary material for: Data on the genome and proteome profiles of ciprofloxacin-resistant Acholeplasma laidlawii strains selected under different conditions in vitro
Source: Data Brief. 2020 Oct 19;33:106412. doi: 10.1016/j.dib.2020.106412 (PMC7585042; doi:10.1016/j.dib.2020.106412)
Supplement: Supplementary file 5 [file mmc5.docx]

**Supplementary table 5.** List of differentially expressed proteins in cells of *A.laidlawii* PG8Bc-3 and *A.laidlawii* PG8r1

| Spot number | Protein name | UniProt^1^ | Score^2^ | n^4^ | Fold^5^ |
| --- | --- | --- | --- | --- | --- |
| 57 | dTTP/UTP pyrophosphatase | A9NE14 | 140 | 11 | 0,5 |
| 82 | 50S ribosomal protein L7/L12 | A9NEL5 | 92 | 7 | 0,57 |
| 19 | Elongation factor Tu | A9NEN4 | 48 | 9 | 1,69 |
| 81 | Sugar-phosphate isomerase, RpiB/LacA/LacB family | A9NEQ1 | 88 | 5 | 0,46 |
| 66 | Superoxide dismutase | A9NEZ4 | 59 | 8 | 4,74 |
| 80 | Molecular chaperone, heat shock protein Hsp20 | A9NFB2 | 136 | 6 | 1,7 |
| 53 | 3-oxoacyl-[acyl-carrier-protein] reductase | A9NFF2 | 181 | 17 | 0,03 |
| 10 | Trigger factor | A9NFM3 | 96 | 18 | 0,06 |
| 63 | Protein GrpE | A9NFN7 | 192 | 18 | 0,31 |
| 9 | Hypothetical surface-anchored protein | A9NFZ8 | 110 | 14 | 0,49 |
| 2 | Polyribonucleotide nucleotidyltransferase | A9NGE2 | 69 | 8 | 0,23 |
| 65 | Uncharacterized protein | A9NGN7 | 44 | 3 | 1,84 |
| 76 | Transcriptional regulator, MarR family | A9NH31 | 140 | 10 | 0,53 |
| 14 | Pyruvate kinase | A9NH70 | 143 | 21 | 0,36 |
| 35 | Glyceraldehyde-3-phosphate dehydrogenase* | A9NHE5 | 65 | 16 | 0,45 |
| 36 | Glyceraldehyde-3-phosphate dehydrogenase* | A9NHE5 | 110 | 21 | 1,86 |
| 43 | SUF system FeS cluster assembly protein | A9NHI6 | 50 | 8 | 0,59 |
| 8 | 60 kDa chaperonin | A9NHL6 | 102 | 13 | 0,2 |

^1^ Identification number of a protein in UniProt database; ^2^ score of protein; ^3^ number of matched peptides; ^4^ fold change in expression compared to control.

* - The identified proteins have different isoelectric points.
