# Supplementary material for: Data on the genome and proteome profiles of ciprofloxacin-resistant Acholeplasma laidlawii strains selected under different conditions in vitro
Source: Data Brief. 2020 Oct 19;33:106412. doi: 10.1016/j.dib.2020.106412 (PMC7585042; doi:10.1016/j.dib.2020.106412)
Supplement: Supplementary file 6 [file mmc6.docx]

**Supplementary table 6.** List of differentially expressed proteins in cells of *A.laidlawii* PG8Bc-3 and *A.laidlawii* PG8r3

| Spot number | Protein name | UniProt^1^ | Score^2^ | n^4^ | Fold^5^ |
| --- | --- | --- | --- | --- | --- |
| 57 | dTTP/UTP pyrophosphatase | A9NE14 | 140 | 11 | 2,35 |
| 59 | Phosphate propanoyltransferase* | A9NE76 | 189 | 14 | 2,51 |
| 60 | Phosphate propanoyltransferase* | A9NE76 | 156 | 13 | 2,73 |
| 64 | Transcription termination/antitermination protein NusG | A9NEJ7 | 93 | 13 | 1,63 |
| 1 | Starvation-inducible DNA-binding protein, ferritin-like protein | A9NEP4 | 70 | 7 | 6,14 |
| 55 | Ribonuclease 3 | A9NES4 | 96 | 9 | 1,94 |
| 66 | Superoxide dismutase | A9NEZ4 | 59 | 8 | 6,19 |
| 22 | Enolase** | A9NF93 | 300 | 26 | 0,13 |
| 23 | Enolase** | A9NF93 | 140 | 17 | 2,06 |
| 24 | Enolase** | A9NF93 | 115 | 14 | 1,91 |
| 78 | Molecular chaperone, heat shock protein Hsp20*** | A9NFB2 | 126 | 10 | 2,16 |
| 79 | Molecular chaperone, heat shock protein Hsp20*** | A9NFB2 | 63 | 5 | 2,38 |
| 80 | Molecular chaperone, heat shock protein Hsp20*** | A9NFB2 | 136 | 6 | 0,37 |
| 53 | 3-oxoacyl-[acyl-carrier-protein] reductase | A9NFF2 | 181 | 17 | 3,18 |
| 39 | Probable endonuclease 4 | A9NFH1 | 86 | 7 | 3,53 |
| 29 | Acetate kinase | A9NFI4 | 151 | 17 | 1,89 |
| 10 | Trigger factor | A9NFM3 | 96 | 18 | 0,48 |
| 63 | Protein GrpE | A9NFN7 | 192 | 18 | 0,64 |
| 7 | Chaperone protein DnaK | A9NFN8 | 76 | 16 | 0,45 |
| 27 | Thymidine phosphorylase | A9NG19 | 133 | 18 | 3,77 |
| 47 | 2-nitropropane dioxygenase-like protein | A9NG76 | 236 | 22 | 3,83 |
| 54 | Cytidylate kinase | A9NGK9 | 311 | 26 | 2,36 |
| 70 | Transcription elongation factor GreA | A9NGN4 | 179 | 12 | 4,31 |
| 65 | Uncharacterized protein | A9NGN7 | 44 | 3 | 3,38 |
| 61 | Ribosome-recycling factor | A9NHC3 | 94 | 11 | 5,86 |
| 35 | Glyceraldehyde-3-phosphate dehydrogenase | A9NHE5 | 65 | 16 | 1,56 |
| 51 | Triosephosphate isomerase | A9NHH5 | 140 | 15 | 2,19 |
| 8 | 60 kDa chaperonin**** | A9NHL6 | 102 | 13 | 27,29 |
| 11 | 60 kDa chaperonin**** | A9NHL6 | 97 | 16 | 4,41 |
| 13 | 60 kDa chaperonin**** | A9NHL6 | 216 | 25 | 0,6 |
| 37 | Pyruvate dehydrogenase E1 component, beta subunit | A9NHS6 | 196 | 21 | 0,48 |
| 30 | Pyruvate dehydrogenase E1 component subunit alpha | A9NHS7 | 134 | 6 | 1,77 |

^1^ Identification number of a protein in UniProt database; ^2^ score of protein; ^3^ number of matched peptides; ^4^ fold change in expression compared to control.

*, **, ***, **** - The identified proteins have different isoelectric points.
